# Occupational-like organophosphate exposure disrupts microglia and accelerates deficits in a rat model of Alzheimer’s disease

**DOI:** 10.1038/s41514-018-0033-3

**Published:** 2019-01-22

**Authors:** Jaymie R. Voorhees, Matthew T. Remy, Claire M. Erickson, Laura M. Dutca, Daniel J. Brat, Andrew A. Pieper

**Affiliations:** 1 Department of Psychiatry, University of Iowa Carver College of Medicine, Iowa City, IA USA; 2 Interdisciplinary Graduate Program in Human Toxicology, University of Iowa Graduate College, Iowa City, IA USA; 3 The Iowa City Department of Veterans Affairs Center for the Prevention and Treatment of Visual Loss, Iowa City, Iowa, United States Departments of Ophthalmology and Visual Sciences, The University of Iowa, Iowa City, IA USA; 4 Department of Pathology, Northwestern University Feinberg School of Medicine, Chicago, IL USA; 5 Harrington Discovery Institute, University Hospital Case Medical Center; Department of Psychiatry Case Western Reserve University, Geriatric Research Education and Clinical Centers, Louis Stokes Cleveland VAMC, Cleveland, OH 44106 USA

**Keywords:** Inflammation, Alzheimer's disease

## Abstract

Occupational exposure to organophosphate pesticides, such as chlorpyrifos (CPF), increases the risk of Alzheimer’s disease (AD), though the mechanism is unclear. To investigate this, we subjected 4-month-old male and female wild-type (WT) and TgF344-AD rats, a transgenic AD model, to an occupational CPF exposure paradigm that recapitulates biomarkers and behavioral impairments experienced by agricultural workers. Subsequent cognition and neuropathology were analyzed over the next 20 months. CPF exposure caused chronic microglial dysregulation and accelerated neurodegeneration in both males and females. The effect on neurodegeneration was more severe in males, and was also associated with accelerated cognitive impairment. Females did not exhibit accelerated cognitive impairment after CPF exposure, and amyloid deposition and tauopathy were unchanged in both males and females. Microglial dysregulation may mediate the increased risk of AD associated with occupational organophosphate exposure, and future therapies to preserve or restore normal microglia might help prevent AD in genetically vulnerable individuals exposed to CPF or other disease-accelerating environmental agents.

## Occupational exposure to toxins may accelerate Alzheimer’s disease

The interaction of genes and environment contributes to Alzheimer’s disease (AD). For example, agricultural workers, military personnel, industrial manufacturers, veterinarians, horticulturists, aircraft maintenance personnel, and pilots are all potentially at risk of occupational exposure to organophosphates (OPs), which are associated with increased risk of AD. We report here that occupational-like exposure of young animals to the OP chlorpyrifos (CPF) accelerates AD-like cognitive deficits and severe neurodegeneration in male, but not female, TgF344-AD rats, a genetic model of AD. CPF exposure also causes chronic dysregulation of brain microglial cells, while amyloid and tau pathology are not affected. Thus, microglial dysregulation after environmental toxin exposure may represent a second hit that advances the disease. Future therapies to preserve or restore normal microglia might help prevent AD in genetically vulnerable individuals exposed to CPF or other disease-accelerating environmental agents.

## Introduction

Synergistic interactions between genes and the environment play a prominent role in major neurodegenerative diseases, including Alzheimer’s disease (AD), Parkinson’s disease, amyotrophic lateral sclerosis, and Huntington’s disease.^1,2^ For example, occupational exposure to organophosphate (OP) pesticides, which inhibit acetylcholinesterase through irreversible covalent modification, increases the risk of AD.^3–7^ In addition to agricultural and horticultural workers subjected to persistent moderate OP exposures over their professional lifetime,^3,7^ veterinarians, soldiers, exterminators, and aircraft personnel are also at risk for occupational exposure. Understanding the underlying mechanisms could help identify new ways to prevent or treat AD in these populations. However, investigation to date has focused narrowly on acute, high-level exposures,^8,9^ rather than the more common moderate and prolonged occupational-like exposure.^10^ To help address this critical gap, we subjected a rat model of AD to a model of occupational chlorpyrifos (CPF) exposure, a commonly applied OP pesticide. We used TgF344-AD rats, which overexpress two mutations that cause AD: APP_SW_ and PS1∆E9. These animals develop amyloid plaques, hyperphosphorylated tau, neuroinflammation, neuronal cell loss, and behavioral deficits akin to human AD patients, rendering them well-suited for preclinical studies.^11–13^ For our environmental model, we applied an established human-derived occupational exposure paradigm that recapitulates characteristic biomarkers and behavioral impairments in a cohort of Egyptian agricultural workers occupationally exposed to CPF.^14–17^ A physiologically based pharmacokinetic/pharmacodynamic model^18^ was used to establish the doses, duration of treatment, and route of administration to inhibit blood ChE activity in rats to levels comparable with those reported in the Egyptian agricultural workers. Accordingly, we exposed 4-month-old male and female TgF344-AD rats and wild-type (WT) littermates to CPF in this manner, and then longitudinally assessed behavior and brain pathology for more than 2 years. We studied both males and females, because epidemiological studies indicate that women are more susceptible than men to AD,^19^ whereas men are more susceptible than women to the deleterious effects of OP exposure.^20–23^

## Results

### Acute plasma cholinesterase inhibition after CPF exposure

We exposed 4-month-old male and female WT and TgF344-AD rats (*n* = 26-31/group) to the early-life occupational-like CPF exposure paradigm and then assessed behavior and pathology at 6, 9, 12, 15, and 24 months of age (Figure S1). Rats were randomly distributed across groups as they were generated in the colony. Behavioral data were collected in an automated and blinded manner. At each time point, a subset of animals was removed for biochemical and pathological analysis. The 21-day daily CPF exposure paradigm was initiated at 4 months of age, as this precedes pathology and behavioral deficits in TgF344-AD rats.^11^

Plasma cholinesterase activity was measured every 7 days throughout the 21-day exposure period and then periodically throughout the rest of the study. Occupational-like CPF exposure acutely inhibited plasma cholinesterase activity similarly in WT and TgF344-AD rats, without impacting body weight (Figure S2). Males exhibited ~50% and 80% plasma cholinesterase inhibition following exposure to CPF at 3 and 10 mg/kg/d, respectively (Figure S2A). By contrast, females exhibited ~75% and 90% plasma cholinesterase inhibition at each respective dose (Figure S2B). Baseline plasma cholinesterase levels in females were roughly twice that of male counterparts, consistent with previous findings from the 1940s of pseudocholinesterase activity in female rats.^24,25^ Importantly, no animals exhibited signs of cholinergic toxicity, such as tremors, secretions, loose stool, or lethargy, in accordance with other studies achieving the same degree of cholinesterase inhibition.^26,27^ By 6 months of age (8 weeks after initial exposure), cholinesterase activity returned to baseline in all groups and remained relatively constant throughout the study (Figure S2A, B), likely due to clearance of CPF from the body, which has previously been reported.^18^

### Cognitive deficits in male, but not female, TgF344-AD rats 2 months after CPF exposure

**Fig. 1:**
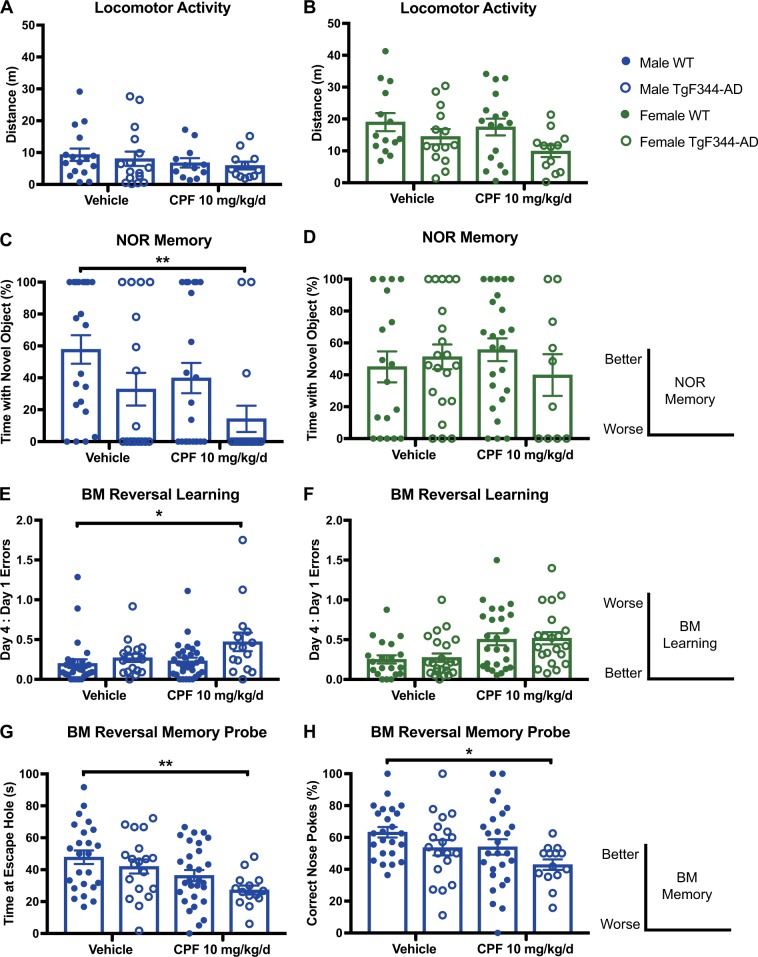
Male TgF344-AD rats are selectively susceptible to accelerated learning deficits as a function of CPF exposure at 6 months of age. **a**, **b** Locomotor activity is not significantly affected by CPF treatment or genotype in males or females at 6 months of age. Open-field treatment effect: male *F*_1,52_ = 1.496, *P* = .2268; female *F*_1,53_ = 1.425, *P* = 0.2379. Open-field genotype effect: male *F*_1,52_ = 0.3095, *P* = 0.5804; female *F*_1,53_ = 5.669, *P* = 0.0209. **c–h** Acquisition of learning and memory deficits in two different tasks is observed in male TgF344-AD CPF-exposed rats, while WT males are unaffected. Female rats of either genotype or exposure group also perform normally in tasks of learning and memory at 6 months of age. For each task, the diagrams on the right indicate the relationship of the magnitude of the measured parameter to quality of performance on the task. **c** TgF344-AD CPF-exposed males display lower preference for the novel object in the NOR task than WT littermates, while there is no statistically significant difference between TgF344-AD and WT male rats in the vehicle group. NOR treatment effect for males was *F*_1,74_ = 3.766, *P* = 0.0561, while NOR genotype effect was *F*_1,74_ = 7.222, *P* = 0.0089. **d** No deficits in females from any group were seen in NOR. NOR treatment effect for females was *F*_1,69_ = 0.001374, *P* = 0.9705, while the NOR genotype effect was *F*_1,69_ = 0.2664, *P* = 0.6074. **e**, **f** Male CPF-exposed TgF344-AD rats make more errors in BM reversal learning, while female CPF-exposed TgF344-AD rats do not. BM reversal learning treatment effect: male *F*_1,86_ = 3.317, *P* = 0.0720; female *F*_1,86_ = 4.576, *P* = 0.0353. BM reversal learning genotype effect: male *F*_1,86_ = 5.972, *P* = 0.0166; female *F*_1,86_ = 0.6869, *P* = 0.4095. **g** Male CPF-exposed TgF344-AD rats spend less time at the escape hole location in the probe test of the BM reversal and **h** make more errors in locating the target in the memory probe phase of the test. No significant impairments in behavior after CPF exposure are observed in females regardless of genotype. BM reversal memory probe treatment effect on time spent at escape: male *F*_1,82_ = 10.04, *P* = 0.0022; female *F*_1,88_ = 2.083, *P* = 0.1525. BM reversal memory probe genotype effect on time spent at escape: male *F*_1,82_ = 3.205, *P* = 0.0771; female *F*_1,88_ = 0.0657, *P* = 0.7984. BM reversal memory probe treatment effect on percent correct nose pokes: male *F*_1,82_ = 4.780, *P* = 0.0317; female *F*_1,86_ = 0.0081, *P* = 0.9283. BM reversal memory probe genotype effect on percent correct nose pokes: male *F*_1,82_ = 5.296, *P* = 0.0243; female *F*_1,86_ = 1.236, *P* = 0.2694. For each task, *n* = 15-31/group. Data are represented as mean ± SEM. Significance was determined using two-way ANOVA with Tukey’s post-hoc multiple comparison analysis. *P < 0.05; **P < 0.01

Throughout the entire study, no significant behavior or pathological effects were observed in the 3 mg/kg/d CPF exposure groups relative to WT controls. Thus, here we present data only from the 10 mg/kg/d CPF exposure group. At 6 months of age, spontaneous open-field locomotor activity was consistent across all treatment groups and genotypes, with females generally traveling more distance than males (Fig. 1a, b). On cognitive tasks of learning and memory, 6-month-old male TgF344-AD rats displayed CPF-dependent deficits that were not seen in WT males or females of either genotype. For example, in the novel object recognition (NOR) memory task, male TgF344-AD rats exposed to CPF spent significantly less time with the novel object, relative to male WT littermates exposed to vehicle (*P* = 0.0098) (Fig. 1c). Although neither CPF-exposed WT animals nor vehicle-exposed TgF344-AD rats showed a significant difference from vehicle-exposed WT rats, CPF-exposed TgF344-AD rats did. We interpret this as a CPF-dependent acceleration of cognitive deficit in the background of the TgF344-AD genotype. Female TgF344-AD rats, by contrast, showed no impairment in the NOR memory task under either vehicle- or CPF-exposed conditions (Fig. 1d). In the learning phase of the Barnes maze (BM) reversal task, male TgF344-AD rats also exhibited deficits relative to WT littermates as a function of CPF exposure (Fig. 1e). Furthermore, male TgF344-AD rats exposed to CPF performed worse than WT littermates exposed to vehicle on two measures of memory during the BM probe test—time spent at the escape hole (Fig. 1g) and percentage of correct nose pokes (Fig. 1h). Female TgF344-AD rats, however, were resistant to CPF-induced learning and memory impairment (Fig. 1f).

### Temporary resolution of CPF-induced cognitive impairment in male TgF344-AD rats at 9 and 12 months of age, and reemergence at 15 months

**Fig. 2:**
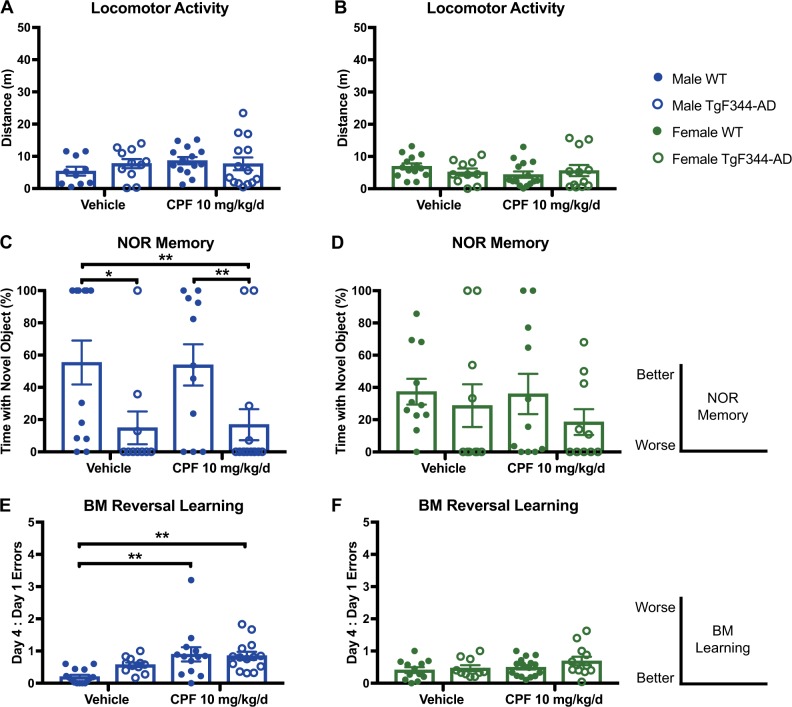
CPF-exposed 15-month-old TgF344-AD rats display sex-dependent, chronic behavioral deficits. **a**, **b** Locomotor activity is not significantly affected by CPF treatment or genotype in males and females at 15 months of age. Open-field treatment effect: male *F*_1,46_ = 0.0603, *P* = 0.8066; female *F*_1,47_ = 0.7429, *P* = 0.3931. Open-field genotype effect: male *F*_1,46_ = 0.0723, *P* = 0.7893; female *F*_1,47_ = 0.0534, *P* = 0.8176. For each task, the diagrams on the right indicate the relationship of the magnitude of the measured parameter to quality of performance on the task. **c–f** Learning and memory deficits are seen specifically in male TgF344-AD rats. **c** TgF344-AD males have a lower preference for the novel object in the NOR task, regardless of CPF exposure. NOR treatment effect for males was *F*_1,43_ = 0.0004, *P* = 0.9846, while the NOR genotype effect was *F*_1,43_ = 10.91, *P* = 0.0019. **e** CPF-exposed WT and TgF344-AD males make more errors in BM reversal learning. BM reversal learning treatment effect for males was *F*_1,49_ = 12.15, *P* = 0.0010, while the BM reversal learning genotype effect was *F*_1,49_ = 1.106, *P* = 0.2980. **d**, **f** No deficits were observed in females regardless of genotype or treatment group. NOR treatment effect for females was *F*_1,38_ = 0.2915, *P* = 0.5924, while the NOR genotype effect was *F*_1,38_ = 0.4810, *P* = 0.2311. BM reversal learning treatment effect for females was *F*_1,46_ = 2.554, *P* = 0.1169, while the BM reversal learning genotype effect was *F*_1,46_ = 1.765, *P* = 0.1906. For each task, *n* = 10–15/group. Data are represented as mean ± SEM. Significance was determined using two-way ANOVA with Tukey’s post-hoc multiple comparison analysis. *P < 0.05; **P < 0.01

At 9 and 12 months of age, respectively 17 and 31 weeks after CPF exposure, males and females in all groups were indistinguishable from WT vehicle controls in locomotor activity, NOR, and BM (Figures S3 and S4). In contrast to these middle-age time points, 15-month-old male TgF344-AD rats (43 weeks after CPF exposure) showed significant cognitive deficits. Although locomotor activity was similar among all groups regardless of exposure, genotype, or sex (Fig. 2a, b), male TgF344-AD rats compared with WT littermates showed significantly reduced preference for the novel object in the NOR task (Fig. 2c). Performance in this task was so poor in 15-month-old male TgF344-AD rats; however, no effect of CPF exposure could be detected (Fig. 2c). Females showed no differences in performance in this task as a function of genotype or CPF exposure (Fig. 2d).

On the BM reversal learning task, male TgF344-AD rats performed significantly worse than WT controls when compared directly in a Student’s *t* test (*P* = 0.004). This learning deficit in TgF344-AD males was exacerbated by CPF exposure (*P* = 0.007) (Fig. 2e). CPF-exposed WT males also performed significantly worse than vehicle-exposed WT males in this task (*P* = 0.003) (Fig. 2e). In females, there were no differences in BM reversal learning as a function of genotype or CPF exposure (Fig. 2f).

### Persistence of CPF-induced cognitive impairment at 24 months in male TgF344-AD rats, and onset of cognitive impairment in age-matched male WT littermates

**Fig. 3:**
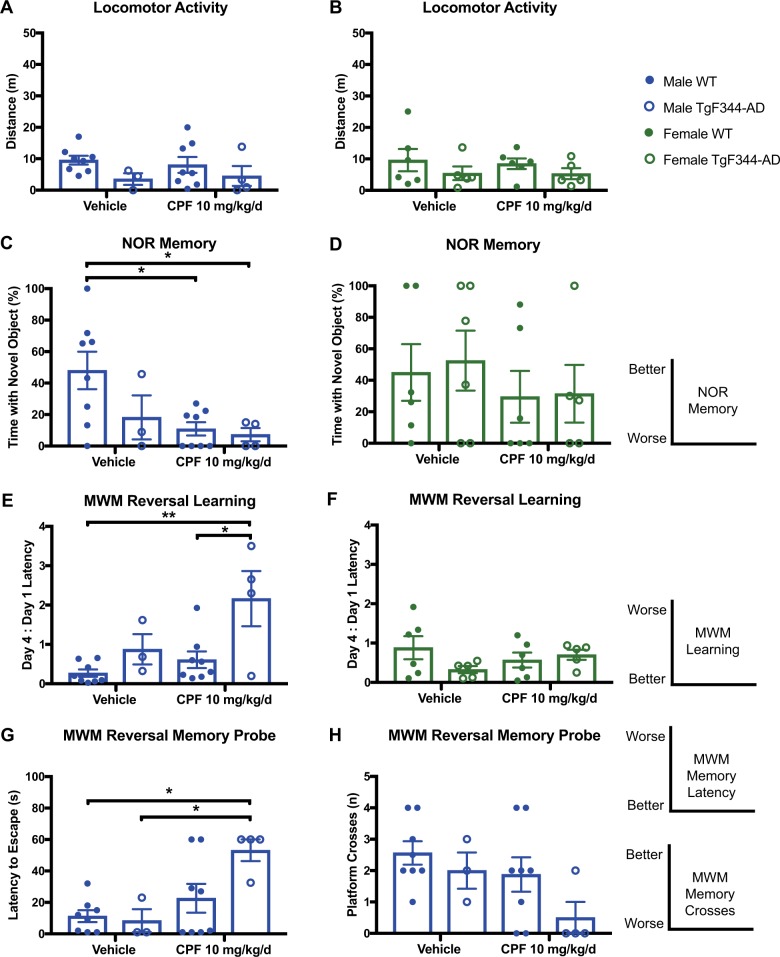
Chronic behavioral deficits in TgF344-AD males following CPF exposure are persistent at 24 months of age. **a**, **b** Locomotor activity is not significantly affected by CPF treatment or genotype in males and females at 24 months of age. Open-field treatment effect: male *F*_1,17_ = 0.0080, *P* = 0.9297; female *F*_1,20_ = 0.1786, *P* = 0.6771. Open-field genotype effect: male *F*_1,17_ = 2.911, *P* = 0.1062; female *F*_1,20_ = 1.450, *P* = 0.2425. For each task, the diagrams on the right indicate the relationship of the magnitude of the measured parameter to quality of performance on the task. **c**–**h** Learning and memory deficits are observed in TgF344-AD males regardless of treatment in both the NOR and MWM task. **c** In the NOR task, WT males exposed to CPF also perform worse than vehicle-exposed WT males. NOR treatment effect for males was *F*_1,19_ = 5.108, *P* = 0.0358, while the NOR genotype effect was *F*_1,19_ = 2.476, *P* = 0.1321. **e** In the MWM reversal task, CPF-exposed TgF344-AD males make more errors learning. MWM reversal learning treatment effect for males was *F*_1,17_ = 5.239, *P* = 0.0352, while the MWM reversal learning genotype effect was *F*_1,17_ = 9.220, *P* = 0.0075. **g**, **h** In the MWM reversal memory probe, CPF-exposed TgF344-AD males spend significantly more time locating the target, and also cross the platform location fewer times. MWM reversal memory probe time to platform treatment effect for males was *F*_1,17_ = 9.726, *P* = 0.0063, while the MWM reversal memory probe time to platform genotype effect was *F*_1,17_ = 2.555, *P* = 0.1284. MWM reversal memory probe platform crosses treatment effect for males was *F*_1,17_ = 3.498, *P* = 0.0788, while the MWM reversal memory probe platform crosses genotype effect was *F*_1,17_ = 2.678, *P* = 0.1201. **d**, **f** Again, no significant behavioral deficits were seen in females regardless of genotype or treatment group. NOR treatment effect for females was *F*_1,19_ = 1.331, *P* = 0.2630, while the NOR genotype effect was *F*_1,19_ = 0.0027, *P* = 0.9593. MWM reversal learning treatment effect for females was *F*_1,18_ = 0.0251, *P* = 0.8758, while the MWM reversal learning genotype effect was *F*_1,18_ = 1.059, *P* = 0.3171. For each task, *n* = 3–8/group (WT vehicle: male *n* = 6, female *n* = 7; TgF344-AD vehicle: male *n* = 3, female *n* = 7; WT CPF 10 mg/kg/d: male *n* = 8, female *n* = 5; TgF344-AD CPF 10 mg/kg/d: male *n* = 4, female *n* = 5.) Data are represented as mean ± SEM. Significance was determined using two-way ANOVA with Tukey’s post-hoc multiple comparison analysis. *P < 0.05; **P < 0.01

At 24 months of age, spontaneous open-field locomotor activity remained unaltered in all groups (Fig. 3a, b). Within the NOR task, CPF-dependent cognitive deficits in TgF344-AD males persisted, and this effect emerged in WT males as well (Fig. 3c). Females, however, showed no differences in any group. To further assess hippocampal-dependent spatial learning and memory at this final time point, we employed the Morris water maze (MWM), to which all animals were naive. The MWM is more sensitive than the BM for hippocampal-dependent spatial learning and memory deficits in rodents.^28^ At the earlier time points, we chose not to conduct repeated MWM testing, despite increased sensitivity, because of its effects on stress hormones^29^ and the role of stress hormones in AD pathology.^30,31^ As the 24-month time point was the final stage of testing, however, we proceeded with MWM. CPF-exposed TgF344-AD males exhibited significant learning and memory deficits at 24 months of age, relative to WT littermates, in the MWM reversal learning and memory probe tests, while CPF-exposed WT males did not (Fig. 3e, g, h). Once again, no CPF-associated cognitive deficits were noted in females of either genotype (Fig. 3d, f).

### Exacerbation of pathological vacuolization and neuronal cell loss in CPF-exposed male and female TgF344-AD rats

**Fig. 4:**
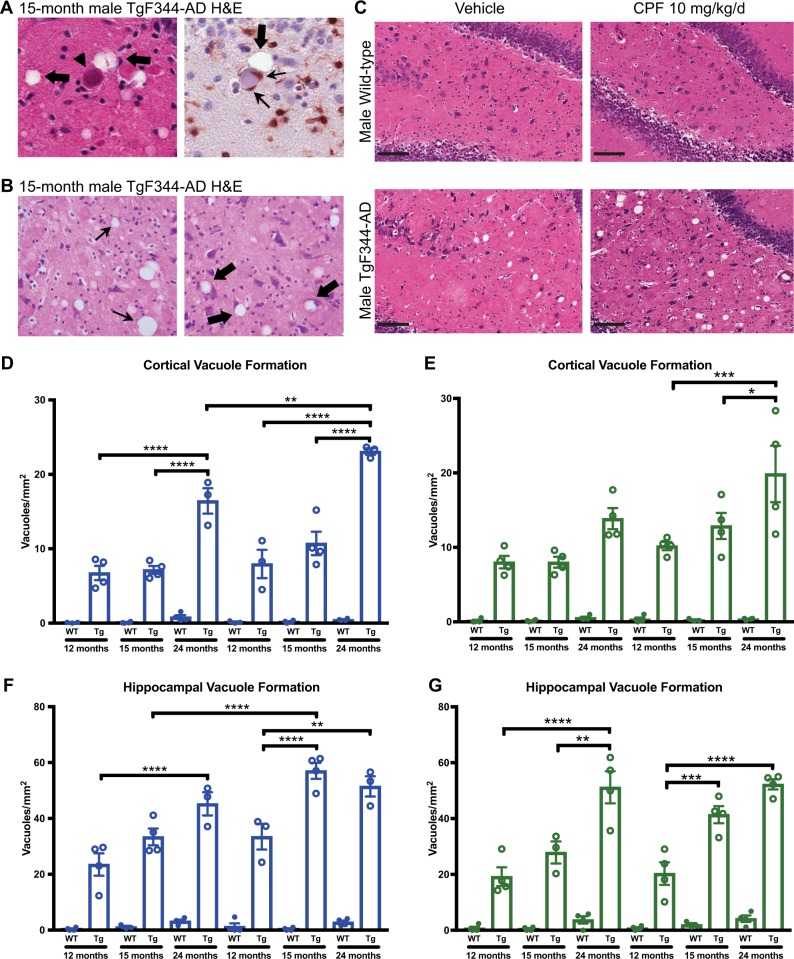
Vacuole formation in both male and female TgF344-AD rats at 15 months of age is exacerbated by CPF exposure. **a** Representative images of vacuoles in the brains of 15-month-old TgF344-AD males. Left: H&E staining reveals vacuoles (identified by thick black arrows) in close proximity with an eosinophilic body center (identified by black arrow head). Right: vacuolization (identified by thick black arrows) is noted in close proximity to a swollen cell body surrounded by Iba1 + cells, a common marker for microglia, likely indicating phagocytosis of neuronal debris (indicated by thin black arrows). **b** Left: representative vacuoles resulting from neuronal cell loss (identified by thin black arrows). Right: representative intracellular vacuoles displacing the cell nucleus (identified by thick black arrows). **c** Representative images of hippocampal vacuole formations in male WT and TgF344-AD vehicle and CPF-exposed groups. The black bar represents 100 μm. **d–f** Manual counting of cortical **d**, **e** and hippocampal **f**, **g** vacuole formation revealed an increase in TgF344-AD rats, relative to WT, which was further exacerbated by CPF exposure in females, and to a greater extent in males. Cortical vacuole counts treatment effect: male *F*_1,31_ = 13.32, *P* = 0.0010; female *F*_1,35_ = 7.763, *P* = 0.0086. Cortical vacuole counts genotype effects: male *F*_1,31_ = 139.5, *P* < 0.0001; female *F*_1,35_ = 52.89, *P* < 0.0001. Hippocampal vacuole counts treatment effect: male *F*_1,32_ = 20.56, *P* < 0.0001; female *F*_1,33_ = 3.182, *P* = 0.0836. Hippocampal vacuole counts genotype effects: male *F*_1,32_ = 158.5, *P* < 0.0001; female *F*_1,33_ = 106.9, *P* < 0.0001. For each measurement, *n* = 3–4/group. Data are represented as mean ± SEM. Significance was determined using two-way ANOVA with Tukey’s post-hoc multiple comparison analysis. *P < 0.05; **P < 0.01; ***P < 0.001; ****P < 0.0001

Morphological analysis of brain tissue using hematoxylin and eosin (H&E) staining showed that aging TgF344-AD rats progressively develop cortical and hippocampal vacuoles (Fig. 4a). These vacuoles, which are pathognomonic of neuronal damage, represent areas in the brain where cells have swollen, died, and been cleared by microglia. Alternatively, vacuoles themselves may propagate cytotoxicity when they form intracellularly and displace nuclei (Fig. 4b). Blinded manual counting of vacuoles revealed that TgF344-AD rats develop significantly more vacuoles than WT controls, as expected (Fig. 4c–g). Furthermore, CPF-exposed male and female TgF344-AD rats developed hippocampal and cortical vacuoles at an accelerated rate compared with vehicle groups (Fig. 4c–g). These two brain regions are critically related to the learning and memory deficits observed at this time point. Notably, this CPF-dependent acceleration of neurodegeneration was more pronounced in TgF344-AD males than females.

### Normal amyloid and tau pathology in CPF-exposed male and female TgF344-AD rats

**Fig. 5:**
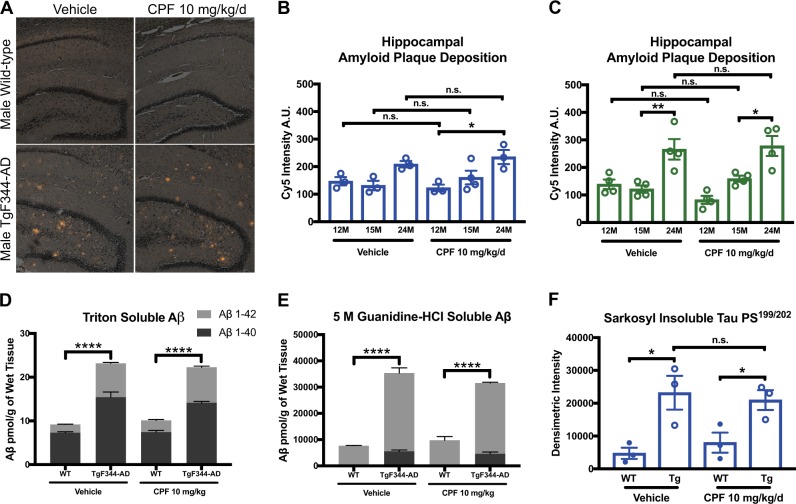
Amyloid deposition and tauopathy in TgF344-AD rats is unaffected by CPF exposure. **a–c** Amyloid plaque deposition is significantly increased in TgF344-AD rats relative to WT rats regardless of CPF treatment. Representative images of Congo red staining in the hippocampus of males are depicted **a**, and male and female hippocampal plaque deposition in TgF344-AD rats is quantified using Cy5 intensity measurements **b**, **c**. Hippocampal plaque deposition treatment effect: male *F*_1,13_ = 0.4062, *P* = 0.5350, female *F*_1,18_ = 0.0113, *P* = 0.9166. Hippocampal plaque deposition genotype effect: male *F*_1,13_ = 11.08, *P* = 0.0016, female *F*_1,18_ = 25.15, *P*<0.0001. **d**, **e** Soluble (triton) and insoluble (5 M guanidine-HCl soluble) Aβ_1–40_ and Aβ_1–42_ peptides are elevated in TgF344-AD rats relative to WT animals at 15 months of age regardless of sex or treatment (males presented here). **f** Pathological tau in the form of hyperphosphorylated insoluble tau is elevated in TgF344-AD rats regardless of sex or treatment (males presented here), as measured by western blot (Figure S7). Total tau protein expression is also elevated in TgF344-AD rats regardless of sex or treatment (presented in Figure S6). Sarkosyl-insoluble abnormally phosphorylated tau protein expression treatment effect for males was *F*_1,8_ = 0.0207, *P* = 0.8893, while the genotype effect was *F*_1,8_ = 20.68, *P* = 0.0019. For each measurement, *n* = 3–4/group. Data are represented as mean ± SEM. Significance was determined using two-way ANOVA with Tukey’s post-hoc multiple comparison analysis. *P < 0.05; **P < 0.01; ****P < 0.0001

Overexpression of mutated APP and mutated PS1 responsible for processing APP leads to amyloid plaque deposition in TgF344-AD rats, a key pathological feature of the brains of AD patients. Initial survey of amyloid plaques in TgF344-AD rats revealed prolific deposition in the prefrontal cortex, cerebral cortex, entorhinal cortex, and hippocampus, with sporadic deposition in the striatum and cerebellum. Plaque deposition progressively increased with age from 12 to 24 months in TgF344-AD groups, but no significant differences at any age in any group were observed as a function of CPF exposure (Fig. 5a–c). Amyloid precursor protein (APP) overexpression was also consistent in TgF344-AD rats regardless of treatment (Figure S5).

In addition to amyloid plaque deposition, abnormal APP processing results in disproportionate formation of amyloid peptides A***β***_1–40_ and A***β***_1–42_. Soluble forms of these peptides are toxic and disrupt synaptic transmission, with A***β***_1–42_ fragments being more toxic than A***β***_1–40_ fragments.^32^ At the 15-month time point, amyloid peptide deposition soluble in triton (i.e., soluble A***β*** peptides) or 5 M guanidine-HCl (i.e., insoluble A***β*** peptides) was further analyzed by A***β***_1–40_ and A***β***_1–42_ peptide ELISAs. Soluble and insoluble A***β***_1–40_ and A***β***_1–42_ were increased in TgF344-AD male and female rats relative to sex-matched controls (Fig. 5d, e), consistent with previous reports.^11^ However, no significant differences were observed as a function of CPF exposure in any animals.

We next assessed expression of tau, a critical microtubule-stabilizing protein in axonal transport. While tau phosphorylation is required for regulation of tau binding to microtubules, hyperphosphorylation of tau in AD results in paired helical filaments that form neurofibrillary tangles (NFTs).^33^ As previously described,^11^ total tau protein expression was elevated in 15-month-old TgF344-AD rats (Figure S6). Furthermore, Tau PS^199/202^ immunoreactivity, a phosphoserine region of tau that is characteristically hyperphosphorylated in AD, was also increased in the sarkosyl-insoluble fraction (NFT formation) in TgF344-AD rats relative to WT controls (Fig. 5f). Total tau and abnormally phosphorylated tau were unaffected by CPF treatment (Figs. S6 and 5f).

### CPF-induced persistent dysregulation of microglia

**Fig. 6:**
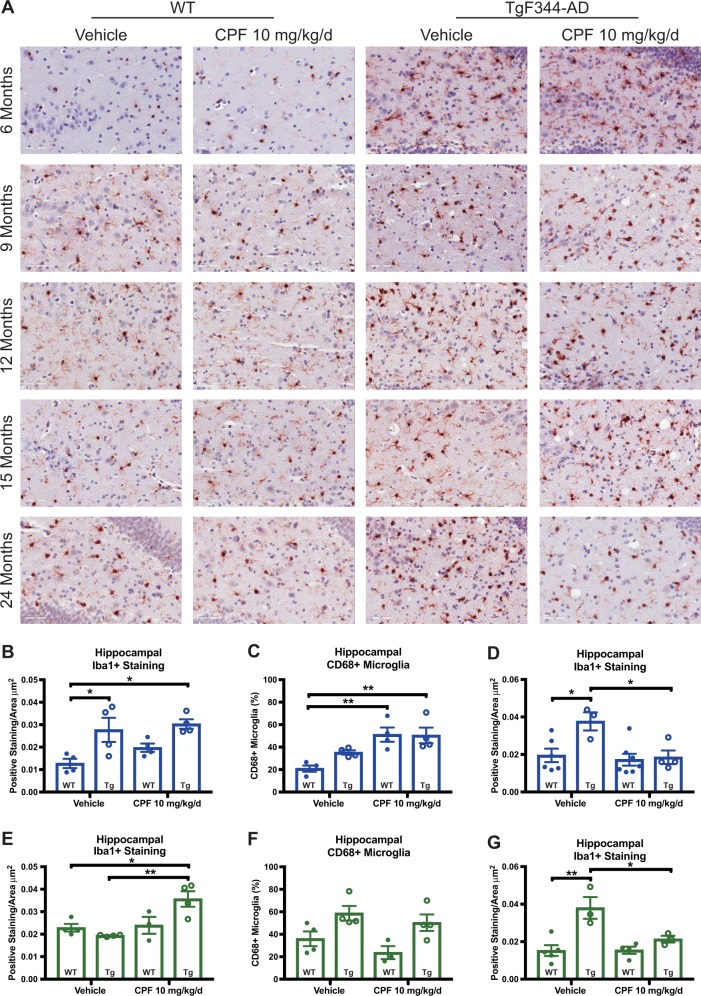
Early-life CPF exposure alters neuroinflammatory responses in TgF344-AD. **a** Representative images of Iba1 + IHC in male WT and TgF344-AD vehicle and CPF-exposed animals at 6, 9, 12, 15, and 24 months of age. At 15 months, Iba1+ stained area in the hippocampus is significantly increased in TgF344-AD rats, and to a greater extent in CPF-exposed male (blue color) **b** and female (green color) **e** TgF344-AD rats. Hippocampal Iba1+ stained area treatment effect at 15 months: male *F*_1,12_ = 2.246, *P* = 0.1598, female *F*_1,11_ = 11.96, *P* = 0.0057. Hippocampal Iba1+ stained area genotype effect at 15 months: male *F*_1,12_ = 15.78, *P* = 0.0019, female *F*_1,11_ = 2.604, *P* = 0.1349. The percentage of CD68+ /Iba1+ cells is also increased at 15 months in TgF344-AD rats, and greatly exacerbated in TgF344-AD CPF-exposed male rats **c** but not female rats **f**. Hippocampal percent CD68 + microglia treatment effect at 15 months: Male *F*_1,12_ = 20.75, p = .0007, Female *F*_1,11_ = 2.320, *P* = 0.1559. Hippocampal percent CD68+ microglia genotype effect at 15 months: male *F*_1,12_ = 1.874, *P* = 0.1962, female *F*_1,11_ = 13.19, *P* = 0.0039. At 24 months, CPF-exposed TgF344-AD microglia populations are significantly depleted in comparison with vehicle-treated TgF344-AD in both males **d** and females **g**. Hippocampal Iba1 + stained area treatment effect at 24 months: male *F*_1,16_ = 7.493, *P* = 0.0146; female *F*_1,11_ = 6.264, *P* = 0.0294. Hippocampal Iba1+ stained area genotype effect at 24 months: male *F*_1,16_ = 6.175, *P* = 0.0244; female *F*_1,11_ = 18.94, *P* = 0.0012. For each measurement, *n* = 3–4/group. Data are represented as mean ± SEM. Significance was determined using two-way ANOVA with Tukey’s post-hoc multiple comparison analysis. *P < 0.05; **P < 0.01

Dysregulation of microglia has been hypothesized to drive histopathology and cognitive decline in AD.^34^ Here, we observed microglial cell population changes in the hippocampus and cortex of male and female TgF344-AD rats (Fig. 6). Staining for the microglial marker ionized calcium-binding adapter molecule 1 (Iba1+) was greater in male but not female vehicle-treated TgF344-AD rats at 15 months of age, relative to WT (Fig. 6b, e). Males and females of both genotypes, however, showed greater predominance of Iba1+ staining as a function of CPF exposure. Female CPF-treated TgF344-AD rats had significantly greater Iba1+ staining relative to vehicle-treated female TgF344-AD rats, while male CPF-treated TgF344-AD exhibited a trending population shift in Iba1+ cells relative to vehicle-treated male TgF344-AD rats. To test whether CPF exposure also rendered microglia more reactive at this time point, we quantified expression of cluster of differentiation 68 (CD68), a marker for microglia reactivity that indicates phagocytosis. The percentage of reactive microglia in CPF-exposed WT and TgF344-AD males was notably increased relative to vehicle-treated controls (Fig. 6c), but this parameter was completely unchanged in females (Fig. 6f). At 24 months of age, 20 months after the onset of CPF exposure, both male and female CPF-exposed TgF344-AD rats exhibited significant reductions in Iba1+ staining in the hippocampus relative to vehicle-exposed TgF344-AD rats, while 20-month-old WT animals of either sex were unaffected by CPF exposure (Fig. 6d, g).

To further interrogate microglial changes, we longitudinally assessed randomized images of Iba1+ immunostaining within the hippocampus and classified microglial morphology into four categories: (1) lowly ramified (0–3 processes), (2) highly ramified (≥4 processes) with large soma, (3) highly ramified (≥4 processes) with small soma, and (5) dystrophic/aged. While microglial morphology changes with age, and TgF344-AD rats displayed distinctly different microglial morphologies from age-matched controls throughout their lifetime, we found no significant differences in morphology as a function of CPF exposure in any animals (Figure S8). Thus, CPF modulates microglia numbers and reactivity, but not morphology. Finally, to determine whether CPF-induced immune responses were localized in the brain or generalized systemically, we measured an array of cytokines, chemokines, and growth factors in the serum of all groups across all time points using a multiplex assay for 14 individual analytes, including IL-6, IL-1α, IL-1β, and TNF-α. There were no significant differences in peripheral blood markers of immune response (Table S1). Thus, sustained changes in immune response following CPF exposure are brain specific.

## Discussion

The goal of this work was to determine whether occupational-like CPF exposure would impact the trajectory of disease development, defined here as the phenotype induced by overexpression of mutant APP and PSEN1. We combined two preclinical models to investigate the interaction between known genetic mutations in AD expressed in TgF344-AD rats and early-life occupational-like exposure to CPF, a commonly applied OP pesticide. Male and female rats of both genotypes exhibited significant inhibition of acetylcholinesterase enzyme activity during the 3-week CPF-exposure period, with enzymatic activity returning to normal 8 weeks later and remaining in normal range thereafter. Two months after completion of CPF exposure, when rats were 6 months old, male TgF344-AD rats exhibited significantly impaired cognitive functioning in the NOR and BM cognitive tasks. Six-month-old TgF344-AD rats in the vehicle control arm of the environmental exposure, however, retained normal cognition. WT littermates also did not manifest cognitive deficits as a function of CPF exposure at this time point. While both NOR and BM tasks measure learning and memory, they employ a unique interplay of different brain regions. For example, NOR utilizes the perirhinal cortex, prefrontal cortex, and hippocampus,^35^ whereas BM uses the entorhinal cortex and hippocampus.^28^ Importantly, the interval of time between behavioral tests was held constant across all animals, in order to increase reliability of the results. Thus, early-life occupational CPF exposure accelerates the onset of broad deficits associated with cortical and hippocampal-dependent tasks of learning and memory in male TgF344-AD rats, but not in WT male littermates or CPF-exposed female rats of either genotype. Notably, the distance traveled in the open field decreased significantly for females in both vehicle- and CPF-exposure groups at all time points after 6 months of age, but this difference was not seen in males. All males and females were handled and aged similarly, and the basis for this interesting sex-specific difference in locomotor activity is not known.

Repeated measures testing in animal behavior can be challenging, and it is important to note that the 9-month-old animals in our study were no longer naive to the BM learning and memory tasks. Thus, it is possible that these animals may have recognized that the escape location had previously rotated, rendering the memory probe less useful. However, learning can still be assessed under these circumstances, because the goal of the task (i.e., finding the escape location) can be achieved and animals remain motivated to find an escape. Furthermore, the NOR test works well for repeated testing, as objects are changed every time. In NOR, memory deficits observed in 6-month-old male CPF-exposed TgF344-AD rats were absent at 9 and 12 months. At 15 months of age, however, the cognitive deficits resurfaced in male TgF344 AD rats. Unfortunately, in the case of NOR, the performance of vehicle-exposed TgF344-AD rats was already so severe that there was no room for detection of possible additive effect of CPF exposure. However, a significant worsening of BM reversal learning was noted in both WT and TgF344-AD male rats as a function of early-life occupational-like CPF exposure. In females, no deficits in either task for either genotype or treatment group were observed.

At 24 months of age, the terminal point of the study, males continued to show susceptibility to cognitive impairment after early-life occupational-like CPF exposure, while females functioned normally. Initially, 10 animals were designated for analysis at the 24-month time point. However, survival in all groups, regardless of sex, genotype, or exposure, was roughly 50%. At this age, ~95% of Fischer rats develop neoplasms that impede mobility or reduce quality of life.^36^ Because repeated surgical mass removal was prohibited by animal care staff, rats were euthanized if they required more than one mass removal. Although the reduced number of animals at the final time point resulted in greater variability, it was clear in the MWM reversal learning phase and memory probe that early-life occupational-like CPF exposure resulted in more greatly impaired cognitive function in TgF344-AD males than in WT male littermates. In general, males appear selectively susceptible to developing cognitive impairment after early-life occupational-like CPF exposure, with exacerbated effect in the presence of genetic susceptibility to AD.

Although female TgF344-AD rats never developed cognitive impairment like males, both sexes displayed cortical and hippocampal vacuolization that was exacerbated by early-life occupational-like CPF exposure. Notably, this effect was more severe in male TgF344-AD rats. Amyloid and tau pathology were not affected by CPF exposure in either sex, but we did observe early microglial proliferation and reactivity as a function of CPF exposure in both males and females, with greater severity in males. Throughout life, a balanced microglia response is critical for maintaining homeostasis in the brain. Under healthy conditions, microglia respond to damage, communicate with neuroinflammatory cells, and clear toxic debris.^37^ Under pathological conditions, however, microglia may become dysregulated such that they do more harm than good, ultimately increasing toxicity and exacerbating neurodegeneration.^37–42^ In our model, microglia in rats with familial AD mutations are dysregulated early in disease. This dysregulation persists chronically and can be exacerbated by environmental insult. Although monocyte populations in TgF344-AD males did not increase in response to CPF treatment, there was a significant shift in the activity state of this cell population toward more phagocytosing. Conversely, females had increased monocyte populations, without a shift in the phagocytosing population in response to CPF exposure. One explanation for this may be that female monocytes increase in number yet remain capable of maintaining a healthy balance of activity, while the males experience a shift in monocyte activity that may advance the disease process.

It is interesting that early-life exposure to CPF induces chronic microglial dysregulation. One explanation may be that microglia undergo a form of cellular senescence following continuous free radical damage, as described by Streit et al.^43^ Future work focused on the effect of CPF on microglia is needed to fully understand the process in this model. Nonetheless, the data are such that it can be proposed that therapeutic strategies aimed at major regulators of phenotypic change of microglia under pathological conditions, such as the TREM2-APOE pathway,^44^ could potentially protect genetically vulnerable populations from disease following microglial-activating environmental events.

Previous studies have described an array of behavioral impairments in WT animals following various exposures to CPF, including deficits in learning, memory, activity, attention, motor coordination, and behavior related to affective state.^10^ Although the goal of this study was to determine consequences of occupational-like CPF exposure in the development of AD-associated behavior and pathophysiology, we also observed interesting effects in aging WT rats. For example, we observed that CPF induces chronic alterations in locomotor activity in WT females, as well as learning and memory deficits in WT males. Furthermore, microglial populations were selectively altered to a more phagocytosing phenotype upon aging in WT males as a function of CPF exposure.

Given that male TgF344-AD rats also showed greater susceptibility to neurodegeneration and resulting cognitive deficits, this male-specific susceptibility may lie within the microglial response to early-life occupational-like CPF exposure. Indeed, Villa et al.^45^ have recently reported significant differences in the transcriptome of male and female microglia from an adult mouse brain, and observed that transplantation of female microglia into adult male mice is neuroprotective in cerebral ischemia. However, alternative hypotheses may also be offered for the lack of behavioral phenotypes observed in female AD- and CPF-exposed groups. For example, we do not know whether exposure levels of CPF differed in the brains of males and females, though we do know that males showed the largest deficit and the least robust inhibition of AchE in the plasma following CPF exposure. In addition, relative to males, females have elevated plasma PON1 activity, a key xenobiotic metabolizing enzyme involved in CPF detoxification that might protect females from CPF-induced neurotoxicity.^21^ It is also possible that the behavior tests employed in this study were not sensitive enough to detect cognitive impairments in females. While numerous measures were taken to reduce confounding factors, including a meticulously designed behavior schedule, separation of sexes throughout behavior testing, and spraying equipment between tests to remove scents, males and females may have reacted differently to the testing environment. Finally, although previous studies have highlighted the effects of ovulation on behavior,^44^ it was not feasible to control for ovulation cycles in our experiments, and we are therefore unable to conclude whether this impacted outcome measures.

It is interesting that no significant behavior effects or pathology were observed in male or female rats exposed to 3 mg/kg/d CPF. This exposure inhibited plasma cholinesterase ~50% (males) and 75% (females), as compared with the 10 mg/kg/d CPF dose that reduced plasma cholinesterase activity by ~75% (males) and 90% (females). An independent study found that 21 days of CPF exposure inhibited brain cholinesterase levels by 50–60% with 3 mg/kg/d and 90% with 10 mg/kg/d, compatible with our observed dose response in neurotoxicity.^15^ This same study also reported significant transcriptomic changes in the hippocampus of animals treated with 10, but not 3, mg/kg/d CPF, using the same CPF-exposure paradigm.^15^

The experimental design employed in this study may prove useful for determining potential links between an array of environmental insults and genetic vulnerabilities to neuropsychiatric disease. We recognize that while the model of early-life occupational-like CPF exposure here is representative of exposures that occur in many countries worldwide, it is not typical of exposures experienced in the USA agricultural industry. Still, global agriculture workers, military personnel, industrial manufacturers, veterinarians, horticulturists, aircraft maintenance personnel, and pilots are all potentially at risk of occupational exposure to OPs, and therefore may also be at an increased risk for developing AD. Our results suggest a new therapeutic direction for protecting these individuals.

## Methods

### Statistical analysis

Data were normally distributed. For multiple mean comparisons, ANOVA was used, followed by post-hoc comparison (Tukey’s method). For direct comparison, two-tailed Student’s *t* test was used. All analyses were conducted using GraphPad Prism (GraphPad Prism Software, Inc, CA). Alpha levels were set to 0.05, and outliers were determined using ROUT method in Prism and removed with Q = 0.1%. Significance is denoted as *P < 0.05; **P < 0.01; ***P < 0.001; ****P < 0.0001. Analyses were blindly conducted. The code was not broken until analyses were completed.

### Study approval

Animal procedures were performed in accordance with University of Iowa Carver College of Medicine Animal Care Committee’s regulations.

### Animals

All animal procedures were performed in accordance with the University of Iowa Office of Animal Resources and Institutional Animal Care and Use Committee (IACUC). We used WT Fischer 344 and heterogeneous TgF344-AD male and female rats on a Fischer 344 background. TgF344-AD rats co-express human APP 695 with mutations (K595N, M596L) and PS1 with a deletion of exon 9 driven on a mouse prion promoter (MPP). A male TgF344-AD breeder was kindly provided by Dr. Robert Cohen and bred with Fischer 344 females from Charles River. All animals were maintained on a 12-h light/dark cycle (6:00 a.m. to 6:00 p.m.) in temperature-controlled conditions (70–72 °F), and animals were provided normal chow (Envigo 7913) and water ad libitum. Animals were housed in a specific pathogen-free (SPF) facility in standard caging (Thoren brand filtered caging) with cellunest bedding (Shepherd Paper Products). Rats were genotyped and given a 4-digit identification number for blinding purposes. Placement into experimental groups was randomly determined at the beginning of the experiment, and all WT controls were littermates. To account for the effects of environmental enrichment on cognition, animals were housed two or three to a cage throughout the duration of the experiment. Occasionally, aging animals required standard veterinary care, and thus, all animal care procedures deemed necessary were performed by a member of the University of Iowa’s Office of Animal Resources veterinary staff according to IACUC regulations. Behavior analysis was performed at least 2 weeks after surgical procedures were performed to reduce confounding factors of anesthesia on behavioral tasks.

### Occupational CPF-exposure model

Four-month-old male and female WT and TgF344-AD rats were randomly placed into treatment groups. Subcutaneous (S.C.) injections of vehicle, 3 mg/kg/d CPF, or 10 mg/kg/d CPF (CAS no. 2921-88-2 obtained from Chem Service, Inc.) were administered between the hours of 8:00–10:00 a.m. daily for 21 days. The vehicle consisted of 10% ethanol and 90% peanut oil. Animals were monitored daily for pain or discomfort. Throughout the exposure period, tail vein blood was collected under isoflurane starting on day one (baseline) and repeated three additional times, 7 days apart, ending on the final day of exposure. Blood was collected in ACD anticoagulant prior to compound administration at each time point, and thus represents circulating levels from the previous day’s administration. Plasma was isolated using a standard practice and samples were stored at −80 °C until further analysis.

### Cholinesterase enzyme activity

Plasma cholinesterase enzyme activity was analyzed using a modified Ellman’s assay.^46^ Briefly, 10 μl of collected plasma was assayed using a Quantichrom Acetylcholinesterase Assay Kit (BioAssay Systems), and samples were analyzed on a Spectromax M2 plate reader at 380 nm. Cholinesterase enzyme activity was normalized to protein concentration, which was measured using a Bradford assay.

### Behavior analysis

At 6, 9, 12, 15, and 24 months of age, animals were subjected to a carefully designed behavior paradigm that consisted of open field, novel object recognition, and Barnes maze (6, 9, 12, and 15 months of age) with reversal task or MWM (24 months of age) with reversal task. Behavior testing was designed to place the most aversive tests at the end of the testing paradigm to reduce confounding factors of consecutive days of testing (Figure S1). For each task, the behavior apparatus was cleaned with 70% ethanol before the first run of the day, between subjects, and after the last run of the day. Furthermore, rats were transported to the experimental testing room in their home cage on the day of each testing session and acclimated to the room 30 min prior to testing.

### Open field

Open-field analysis was used to assess locomotor activity. The open-field apparatus (University of Iowa Medical Shop) was a black, open topped Plexiglas box, measuring 100 × 100 × 30 cm. Rats were placed in the center of the open-field apparatus and allowed to freely explore for 10 min for each test. ANY-Maze video tracking software (Stoelting Co.) was used to measure the distance traveled by each animal, and analysis was conducted blind to treatment group.

### Novel object recognition

A novel object recognition test was performed to assess recognition memory.^47^ and the open-field apparatus (previously described) was used during object recognition analysis. Objects were made of plastic, ranged from 3 to 6 cm in size, and were of different shapes in different colors. NOR testing was completed on animals up to five different times, and thus, different objects were used for each round of repeated testing to maintain novelty in this task. A total of 10 different objects were used throughout the duration of this experiment. Dim lighting was provided in all stages of the testing. Open-field analysis (as previously described) was preformed one day prior to familiarization testing to allow the animal to habituate to the testing apparatus. On the second day of NOR testing (the familiarization phase), rats were placed in the open-field box containing two identical objects (A1 and A2) fixed to the floor and allowed to freely explore the apparatus for 10 min. On the third and final day of NOR testing, the choice phase of the task was performed. Two new objects (A3 and B) were fixed to the floor. Object A1, A2, and A3 were identical, but different from object B, the novel object. The A3 object was used to eliminate any potential for smells from the familiarization to influence the choice phase of the task. Videotape recordings of the familiarization and choice phases were analyzed independently by two blinded scorers, who recorded the time each rat explored each of the objects during the familiarization and choice phases. The percentage of time spent exploring the new object (B) during the choice phase, corrected for any location preference during the familiarization phase, was taken as a measure of memory. Exploration of an object was defined as when the rat directed its nose toward the object at a distance of < 5 cm.

### Barnes maze

Barnes maze testing was conducted to assess learning and memory at 6, 9, 12, and 15 months of age.^48^ The Barnes maze apparatus was a white, circular surface, 120-cm in diameter, and was raised to a height of 40 cm. The maze consisted of 20 holes, 10-cm in diameter, equally spaced around the border of the surface with an escape cup placed under one hole (University of Iowa Medical Shop). Four, high-contrast images, placed equal distance around the apparatus and 10 cm from the top of the table, were used as spatial cues. Over the course of 20 months of behavior testing, 12 different high-contrast images were used as spatial cues. Bright lighting above the apparatus was used to motivate the animals to find an escape location. Each test animal was subjected to 4 days of training comprised of four trials per day, spaced a minimum of 20 min apart, and the time spent to find the escape location and the number of errors (nose pokes in incorrect holes) committed prior to finding the escape cup were recorded for each trial and averaged for each day. The trial ended when the rat entered the escape cup or after 80 s. On day 5, a probe trial was conducted, during which the escape cup was removed and the animal’s memory of the escape location based upon spatial cues was analyzed. ANY-Maze video tracking software (Stoelting Co.) was used to measure the percent time spent in the target area and the percent time spent in the target quadrant. The ratio of the number of nose pokes in the target hole to the number of nose pokes in incorrect holes was manually scored. Nose pokes were defined as head deflections into a hole, but successive pokes into the same hole were not counted as multiple nose pokes. An area extending 5 cm from the escape hole in all directions was used as the target area for measurements.

### Reversal Barnes maze

Reversal testing was used to further assess learning and memory. Following initial Barnes maze analysis, rats were given 2 days of rest and then tested for learning and memory, as well as cognitive flexibility. Accordingly, the escape hole was rotated 180° and associated with a new spatial cue. Again, each animal was given time to explore the Barnes maze platform freely for 80 s or until they located the new escape location. Four trials were conducted each day at a minimum of 20 min apart for 3 days, and the latency to find the escape and the number of errors committed prior to finding the correct escape location were recorded to test the animal’s capacity to learn the escape location. On day 4, the escape cup was removed to test the animal’s memory and the probe assay was scored as previously described.

### Morris water maze

The MWM test was conducted to analyze learning and memory in 24-month-old rats.^49^ The MWM was a silver, circular water tank, measuring 167-cm in diameter, filled with 24–25 °C water to ~60 cm. A clear, plastic platform was placed submerged below the surface of the water in association with one of four high-contrast spatial cues located around the maze. Each test animal was subjected to 4 days of training comprised of four trials per day a minimum of 20 min apart, and the time spent to find the platform or 120 s (if the platform was not located) was recorded for each trial and averaged for each day. On day 5, a probe trial was conducted, during which the platform was removed and the animal’s memory of the escape location based upon spatial cues was analyzed. ANY-Maze video tracking software (Stoelting Co.) was used to measure the latency to and the number of times the animal crossed the previous platform location area.

### Reversal MWM

Reversal testing was used to further assess learning, memory, and cognitive flexibility. Accordingly, the escape platform was rotated 180° and associated with a new spatial cue. Again, each animal was placed in the water tank until they found the location of the platform or for 120 s. Four trials were conducted each day for 3 days, and latency to find the platform was recorded to test the animal’s capacity to learn the escape location. On day 4, the platform was removed to test the animal’s memory and the probe assay was scored as previously described.

### Reagents

Chlorpyrifos (CAS no. 2921-88-2) 98% purity was purchased from Chem Service and diluted in 10% ethanol and 90% peanut oil (Sigma). Paraformaldehyde (PFA) was purchased from Electron Microscopy Sciences and diluted to working concentrations in PBS. An antibody against APP (APP C-terminal) obtained from ThermoFisher Scientific was used at dilutions of 1:1000 for western blot (WB). An antibody against abnormally phosphorylated tau (clone tau-ps199/202) was obtained from Sigma and used at a concentration of 1:1000 for immunohistochemistry (IHC) and 1:4000 for WB. An antibody against Iba1 (1:5000 for IHC) was obtained from Wako Ltd., and an antibody against CD68 (1: 1000 for IHC) was obtained from Abcam. β-Actin (1:1000 for WB) was obtained from Santa Cruz. Secondary antibodies against mouse and rabbit (1:5000–1:10,000 for WB) were obtained from Cell Signaling, and secondary ImmPRESS IgG HRP and ImmPACT Nova Red and DAB kits were obtained from Vector. Congo Red was obtained from Sigma.

### Tissue preparation

Transcardiac perfusions with ice-cold PBS were performed while animals were deeply anesthetized under isoflurane and brains were quickly collected and separated into hemispheres. The left hemisphere was fixed in 4% PFA for 24 h prior to routine processing and paraffin embedding for histochemical analyses. The right hemisphere was snap frozen in liquid nitrogen and stored at −80 °C until further processing. Upon processing, snap-frozen hemispheres were homogenized in 2 mL of ice-cold lysis buffer (Cell Signaling Technology) supplemented with 1 mM PMSF (Sigma), phosphatase inhibitors, and protease inhibitors (Halt ThermoScientific) for biochemical analysis. Briefly, brains were mechanically dissociated using a tissue tearor homogenizer for 10 pulses. Samples were allowed to stand for 15 min at 4 °C and then separated into three equal aliquots and stored at −80 °C until further processing. One aliquot was used for tau analysis, another for Aβ analysis, and the third was used for protein analysis. For tau analysis, the aliquot was centrifuged at 27,000*g* for 20 min at 4 °C and the supernatant was set aside. Pellets were rehomogenized in 3 × (tissue/volume) buffer supplemented with 10% sucrose and salts and then centrifuged at 27,000*g* for 20 min at 4 °C. The supernatant was combined with the first supernatant and the pellet was saved for WB analysis (crude pellet). To isolate sarkosyl-insoluble tau, the combined supernatants were incubated for 1 h at 37 °C in 1% sarkosyl and 1% β-mercaptoethanol. The samples were centrifuged at 150,000*g* for 35 min at room temperature. The resulting pellet (sarkosyl-insoluble P3) was analyzed via WB. For Aβ analysis, the aliquot was centrifuged at 10,000*g* for 15 min at 4 °C. The supernatant (soluble fraction) was centrifuged at 16,000*g* for 20 min at 4 °C and the resulting supernatant was diluted in cell lysis buffer for further analysis. The pellet from the initial centrifugation was rehomogenized in 4 × (tissue/volume) 5 M guanidine-HCl for further analysis.

### Immunohistochemistry and microscopy

Ten-micrometer para-median sagittal sections were sliced at 30-μm intervals using a microtome and mounted on glass slides, beginning approximately at lateral 0.6 mm and extending approximately through lateral 3.25 mm. For histological analysis, three slides containing a total of nine sections beginning at lateral 0.6 mm and spanning 150 μm were analyzed for each subject. The entire cortex, encompassing the angular insular (AI), somatomotor areas (MO), somatosensory areas (SS), posterior parietal association areas (PTLp), and visual areas (Vis), was analyzed. Sections were routinely dewaxed, hydrated in a graded series of ethanol, and placed in a steamer for 25 min in Citra + buffer (R&D) for antigen retrieval. Endogenous peroxidase was quenched with 0.6% H_2_O_2_ for 15 min. Sections were blocked in normal horse serum (Vector) for 20 min, hybridized with various primary antibodies for 1 h, and then incubated with appropriate ImmPRESS secondary kits (Vector). Sections were developed with Nova Red or DAB (Vector) and counterstained with hematoxylin. Routine dehydration in a graded series of ethanol and xylene was preformed prior to coverslipping (Surgipath MM24 Leica). For amyloid burden, sections were directly stained with Congo red according to the manufacturer’s recommendations and imaged using Cy5 fluorescent excitation.^50^ Fluorescent images were obtained using a Zeiss Axiolmager.M2 microscope and quantified using contrast intensity methods in ImageJ. IHC-stained slides were scanned using an Aperio ImageScope and analyzed using Aperio Digital Slide Studio software (Aperio Group LLC, Sausalito, CA, USA). Individually optimized NuclearV9 algorithms were used for Iba1+ and CD68+ cell counts. Individually optimized ColorDeconvolutionV9 algorithms were used to assess Iba1+ stained area.

### Morphological analysis

For morphological analysis, para-median sagittal 10-μm sections, mounted three per slide, spaced 150-μm apart were stained with H&E (ThermoScientific) using routine protocols. Again, sections were imaged using an Aperio ImageScope and analyzed using Aperio Digital Slide Studio software (Aperio Group LLC, Sausalito, CA, USA). Representative sections were subsequently assessed by a neuropathologist. Vacuoles were manually counted from every section and normalized to area analyzed for each region of interest.

### Biochemical analysis

Biochemical analysis of Aβ peptides was conducted according to a two-step extraction method.^51^ Briefly, detergent-soluble Aβ_1–40, 42_ species were separately detected in rat brain homogenates prepared with cell lysis buffer described above at a 1:25 dilution. Detergent-insoluble Aβ_1–40, 42_ species were detected by extraction of homogenate pellets in the chaotropic agent, 5 M guanidine-HCl, followed by a 1:12,500 dilution in lysis buffer. Protein levels were normalized by BCA protein assay (Pierce Biotechnology). Aβ species were separately quantified in detergent-soluble and -insoluble (5 M guanidine-HCl-extracted) fractions using Aβ_1–40, 42_ ELISA kits (Wako Ltd.) in accordance with the manufacturer’s instructions. For tau analysis, pellets were rehomogenized in a 10% salt-sucrose solution to obtain various soluble and insoluble fractions for western blot.^52^ Crude pellet tau was obtained by re-homogenization of pellets with TBS (pH 7.4), followed by centrifugation at 1000*g* for 5 min at 4 °C. 5ein were electrophoretically separated using 4–20% tris-glycine gels. Electrophoresed proteins were then transferred to PVDF membranes, blocked in TBS containing 5% (w/v) BSA (Sigma), and subsequently hybridized with various primary antibodies. Membranes were then incubated with the appropriate HRP-conjugated secondary antibody prior to development with chemiluminescent substrates. ImageJ software was used for densitometric analysis of blots. All blots derive from the same experiment and were processed in parallel.

### Microglia morphological analysis

For morphological analysis of microglia, three Iba1-stained sections were imaged using randomized stereological techniques in the hippocampus, using a three-dimensional counting frame of 300 × 200 × 10-μm on a 500 × 500-μm grid. Positively stained cells were characterized from every section, and percentages of characterized cells were calculated from the total number of cells assessed. For the 9-, 12-, and 15-month time points, greater than 75 cells were analyzed per section. For the 6- and 24-month time points, greater than 50 cells were analyzed per section. The morphology of microglia was classified according to four categories, as modified from Diz-Chaves et al.^53^ (1) lowly ramified (0–3 processes), (2) highly ramified (≥4 processes) with a large soma, (3) highly ramified ( ≥ 4 processes) with a small soma, and (5) very highly ramified (>5 processes) with a large soma and usually described as dystrophic, aged, or bushy microglia.

### Serum analyte analysis

Serum was collected at 6, 9, 12, 15, and 24 months of age using standard protocols, and samples were stored at −80 °C until analysis. Samples underwent a single freeze–thaw cycle for analysis. Analytes in serum were measured using a Th Complete 14-Plex Rat ProcartaPlex™ Panel (Invitrogen; Lot: 151908006) according to the manufacture’s recommendations. Neat (undiluted) serum preparations from all treatment groups at all time points (*n* = 4/group) were analyzed using a BioRad Luminex 200 Flow Cytometer system. Fluorescence intensity was measured and data analyzed using Bio-Plex Data Analysis software.

## Supplementary information

Supplementary Information Figures and Tables

## Data Availability

The datasets generated during and/or analyzed during the current study are available from the corresponding author on reasonable request.

## Supplementary information

**Supplementary information** accompanies the paper on the *npj Aging and Mechanisms of Disease* website (10.1038/s41514-018-0033-3).
