# Supplementary material for: Occupational-like organophosphate exposure disrupts microglia and accelerates deficits in a rat model of Alzheimer’s disease
Source: NPJ Aging Mech Dis. 2019 Jan 22;5:3. doi: 10.1038/s41514-018-0033-3 (PMC6342990; doi:10.1038/s41514-018-0033-3)
Supplement: Supplementary file 1 — Supplementary Information Figures and Tables [file 41514_2018_33_MOESM1_ESM.pdf]

Supplementary Fig 1.

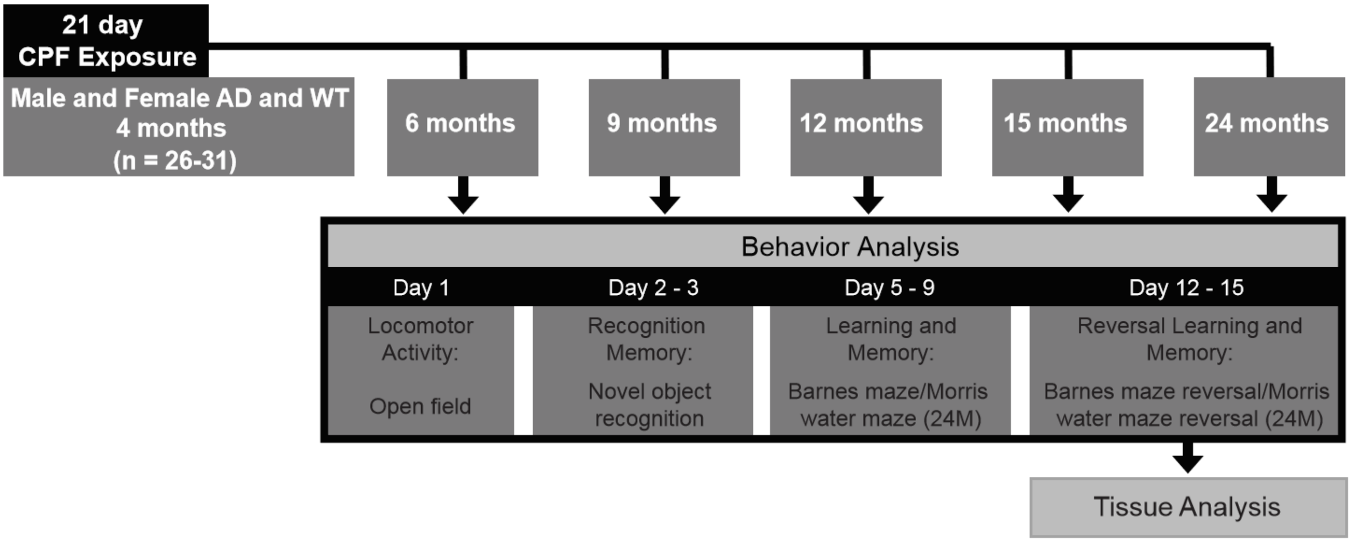

Fig. S1 Experimental Design At 4 months of age, male and female TgF344-AD rats and WT littermates (n = 26-31/group) were exposed for 21 days to 3 or 10 mg/kg/d CPF or vehicle via S.C. injection. Throughout exposure, weight and cholinesterase enzyme activity were measured. At 6, 9, 12, 15, and 24 months of age, the following behaviors were assessed: locomotor activity (open field), recognition memory (novel object recognition), and hippocampal-dependent spatial learning and memory (Barnes maze and Morris water maze). Behavior testing was conducted in a specific order designed to reduce the confounding stress of multiple days of testing, with the least stressful tasks assayed first and the most stressful tasks assayed last. At each time point, a subset of animals was removed for histochemical and biochemical analysis.

Supplementary Fig 2.

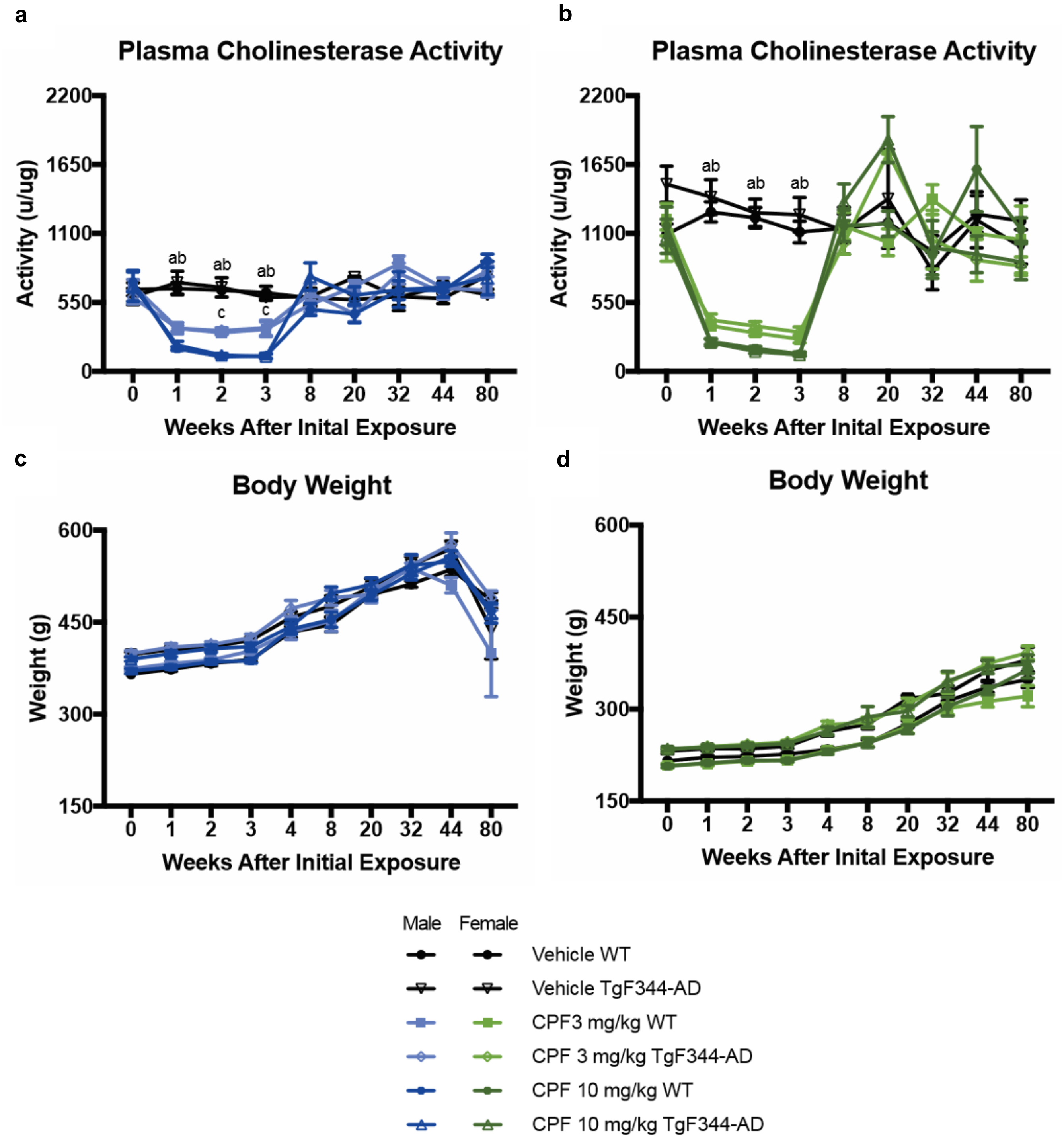

Fig. S2 CPF exposure inhibits plasma cholinesterase but does not affect body weight. (**a-b**) **Plasma cholinesterase enzyme activity was significantly inhibited by both 3 and 10 mg/kg/d CPF by ~50% and 75% respectively in males, while greater inhibition was observed in females (~75% and 90% for 3 and 10 mg/kg/d, respectively).** Plasma cholinesterase levels returned to baseline levels by 8 weeks following the first day of exposure and remained at baseline thereafter. (**c-d**) **Weight was unaffected by exposure or genotype throughout the entire study.** Data are represented as mean ± SEM. Significance was determined using two-way ANOVA with Tukey's post-hoc multiple comparison analysis. ab = $p < .0001$ relative to both 3 and 10 mg/kg/d treatment groups; c = $p < .05$ relative to 10 mg/kg/d treatment group.

Supplementary Fig 3.

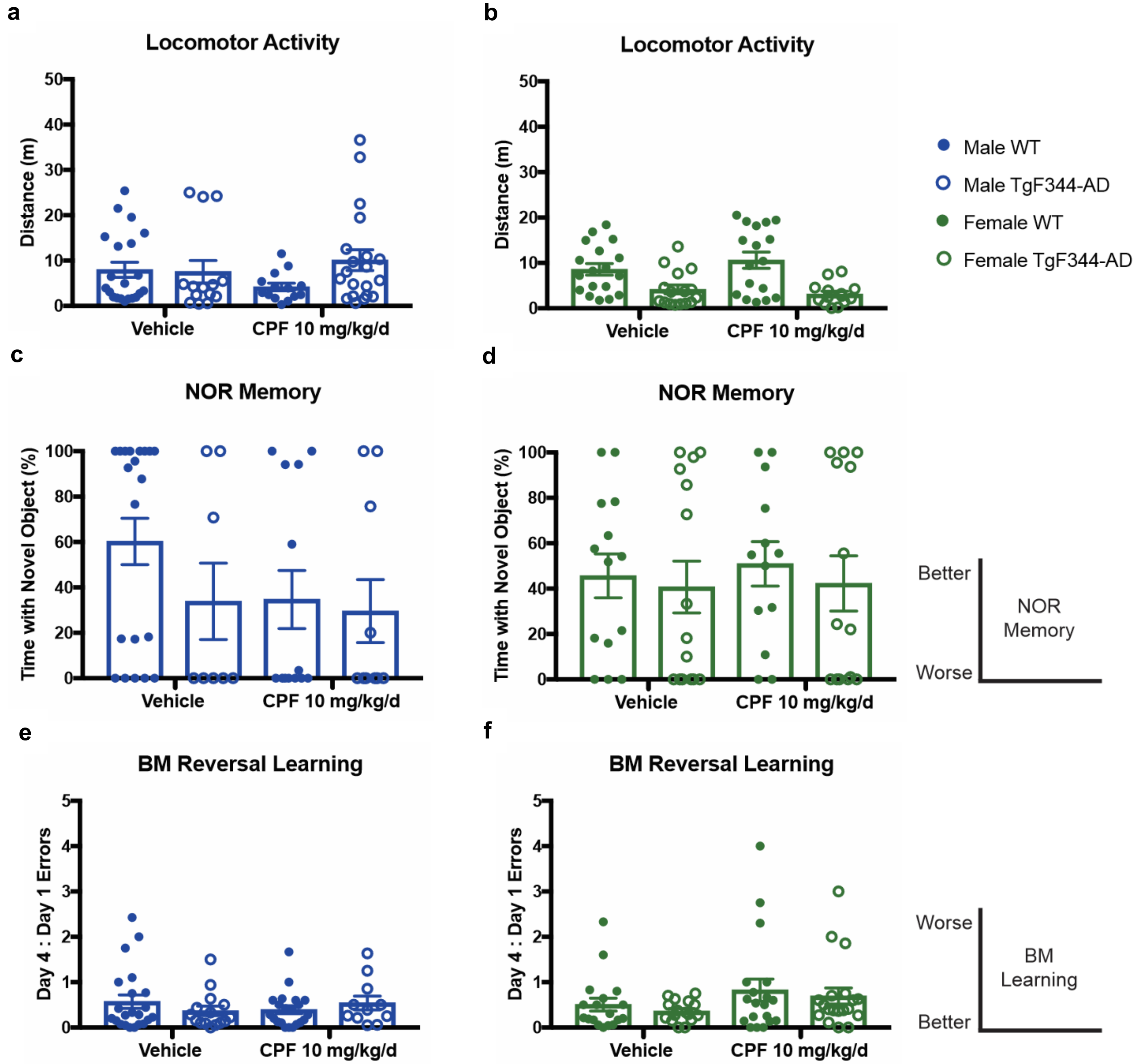

Fig. S3 Neither male of female CPF-exposed TgF344-AD rats display behavioral deficits at 9 months of age. (**a-b**) **Locomotor activity is not significantly affected by CPF treatment or genotype in males and females at 9 months of age.** Open field treatment effect: Male $F_{1,65}$ = 0.0931, p = .7612; Female $F_{1,63}$ = 1.761, p = .1893. Open field genotype effect: Male $F_{1,65}$ = 1.840, p = .1797; Female $F_{1,63}$ = 5.655, p = .0205. For each task, the diagrams on the right indicate the relationship of the magnitude of the measured parameter to quality of performance on the task. (**c-f**) **No significant learning or memory deficits were observed in males or females of any genotype or treatment group at 9 months of age, as measured by NOR (c-d) and BM reversal (e-f).** NOR treatment effect: Male $F_{1,47}$ = 1.212, p = .2765; Female $F_{1,52}$ = 0.1008, p = .7522. NOR genotype effect: Male $F_{1,47}$ = 1.350, p = .2512; Female $F_{1,47}$ = 0.3854, p = .5375. BM reversal learning treatment effect: Male $F_{1,65}$ = 0.0001, p = .9917; Female $F_{1,68}$ = 3.448, p = .0677. BM reversal learning genotype effect: Male $F_{1,65}$ = 0.0438, p = .8350; Female $F_{1,68}$ = 0.6149, p = .4357. For each measurement, n = 9-26/group. Data are represented as mean ± SEM. Significance was determined using two-way ANOVA with Tukey's post-hoc multiple comparison analysis. *p < .05; **p < .01.

Supplementary Fig 4.

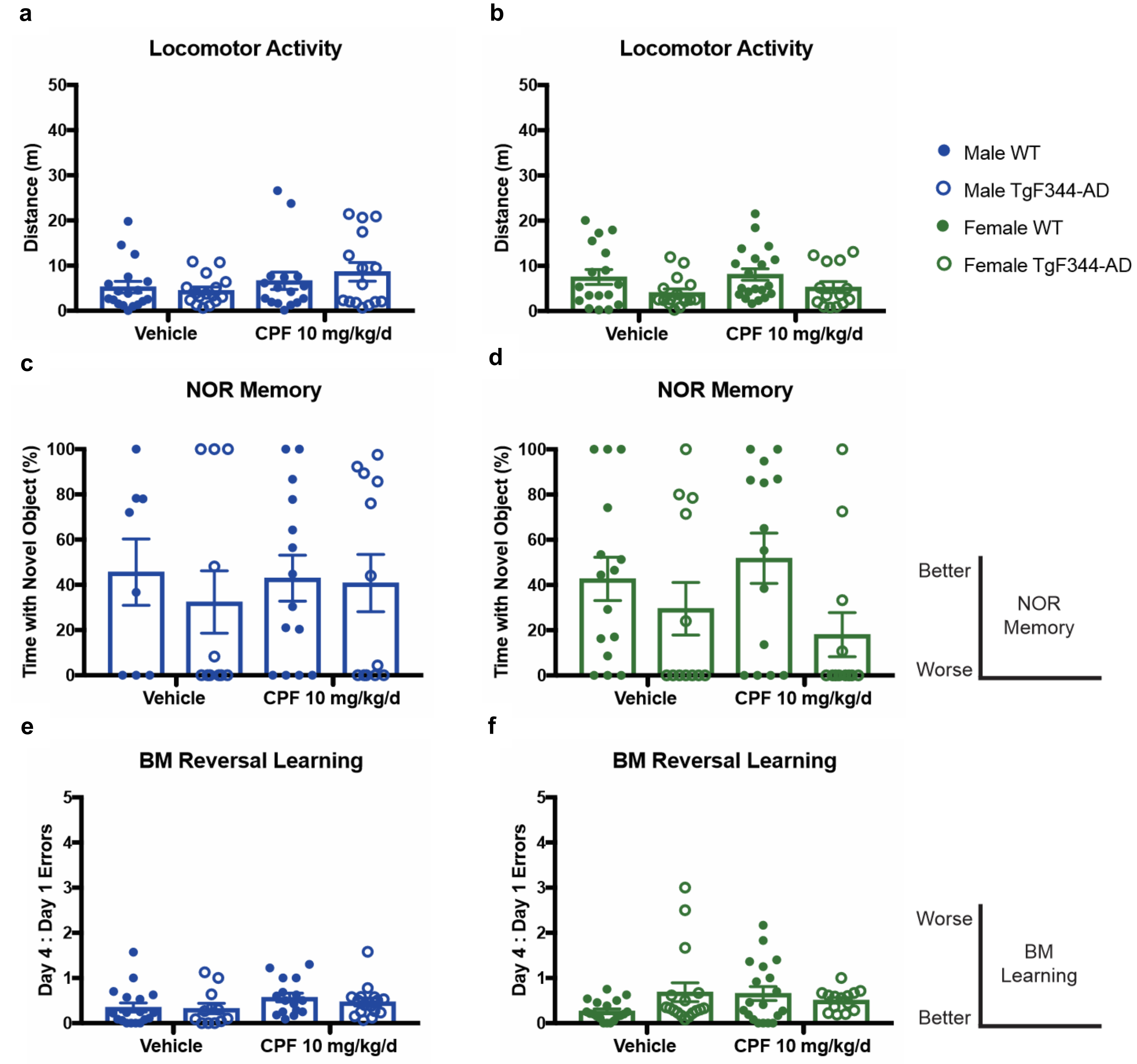

Fig. S4 Neither male or female CPF-exposed TgF344-AD rats display behavioral deficits at 12 months of age. (a-b) Locomotor activity is not significantly affected by CPF treatment or genotype in males and females at 12 months of age. Open field treatment effect: Male $F_{1,61}$ = 3.171, p = .0799; Female $F_{1,63}$ = 0.4630, p = .4987. Open field genotype effect: Male $F_{1,61}$ = 0.1512, p = .6988; Female $F_{1,63}$ = 5.749, p = .0195. For each task, the diagrams on the right indicate the relationship of the magnitude of the measured parameter to quality of performance on the task. **(c-f) No significant learning and memory deficits were observed in males or females of any genotype or treatment group at 12 months of age, as measured by NOR (c-d) and BM reversal (e-f).** NOR treatment effect: Male $F_{1,41}$ = 0.0501, p = .8240; Female $F_{1,49}$ = 0.0123, p = .9121. NOR genotype effect: Male $F_{1,41}$ = 0.3604, p = .5516; Female $F_{1,49}$ = 4.922, p = .0312. BM reversal learning treatment effect: Male $F_{1,58}$ = 3.262, p = .0761; Female $F_{1,65}$ = 2.357, p = .1294. BM reversal learning genotype effect: Male $F_{1,58}$ = 0.3786, p = .5407; Female $F_{1,65}$ = 0.1984, p = .6574. For each measurement, n = 9-20/group. Data are represented as mean ± SEM. Significance was determined using two-way ANOVA with Tukey's post-hoc multiple comparison analysis. *p < .05; **p < .01.

Supplementary Fig 5.

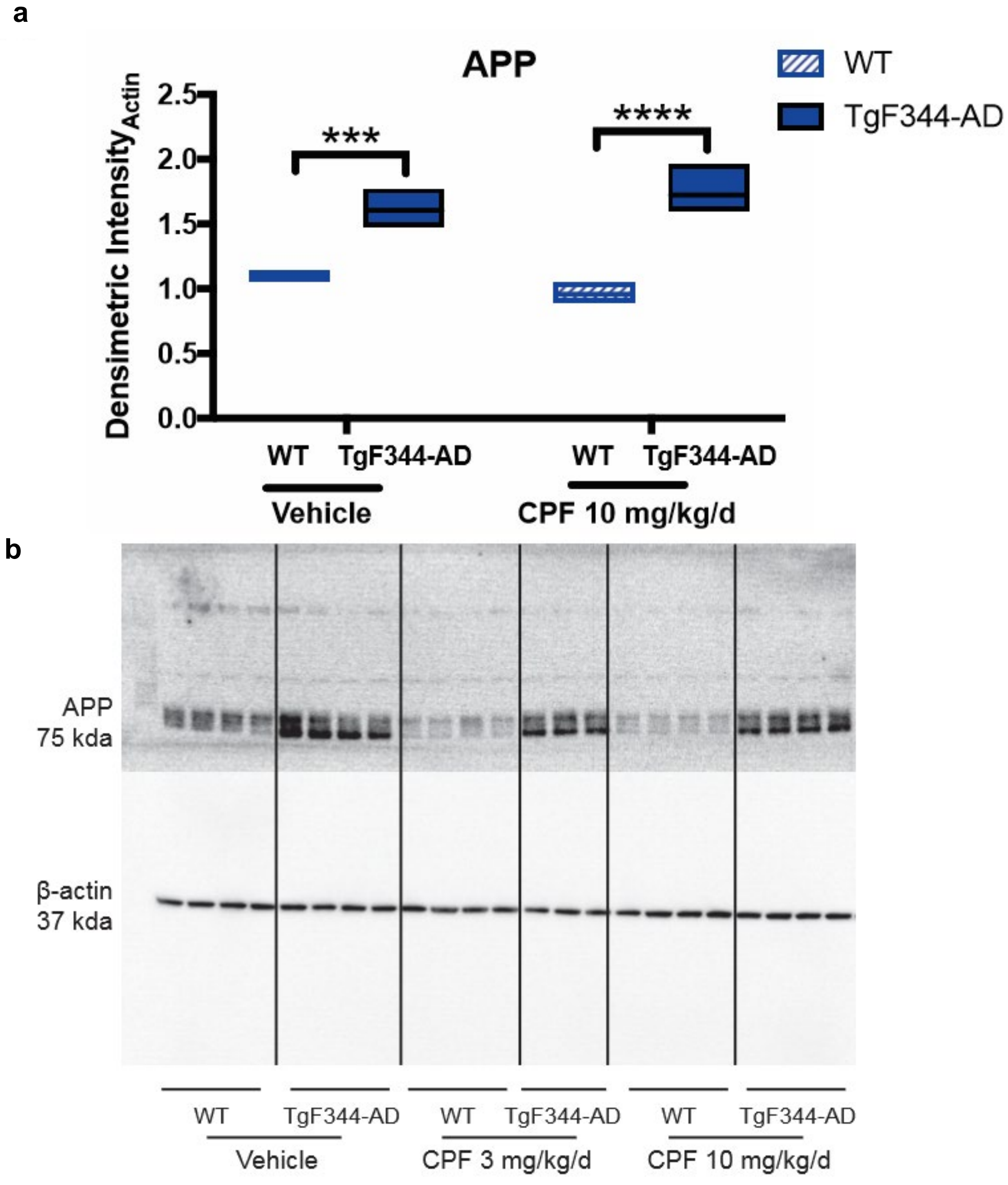

Fig. S5 APP protein expression is elevated in TgF344-AD rats regardless of CPF-exposure. APP expression is elevated in TgF344-AD rats and not affected by CPF exposure (quantified in a), as demonstrated in the representative western blot with densimetric intensity quantified relative to β-actin (b). APP protein expression treatment effect for males was $F_{1,12} = 0.4679$, p = .8324, while the genotype effect was $F_{1,12} = 142.3$, p < .0001. ***p< .001; ****p < .0001.

Supplementary Fig 6.

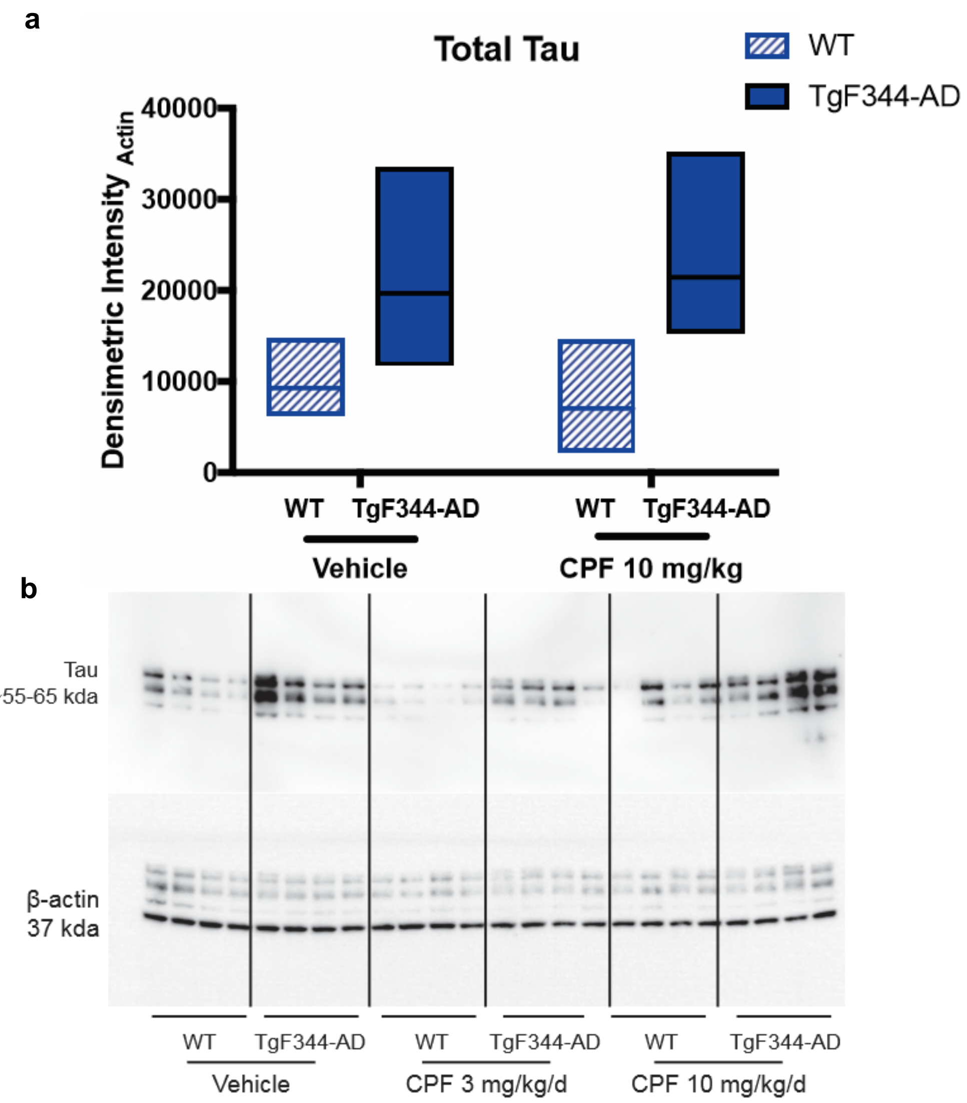

Fig. S6 Total tau protein expression is not significantly elevated in TgF344-AD rats as compared to WT rats regardless of CPF-exposure. WT and TgF344-AD total tau protein is quantified (a) using western blot analysis (b) in rats treated with vehicle or CPF 10 mg/kg/d. Protein levels are normalized to β-actin. Total tau protein expression treatment effect for males was $F_{1,12}$ = 0.0032, p = .9556, while the genotype effect was $F_{1,12}$ = 10.64, p = .0068.

Supplementary Fig 7.

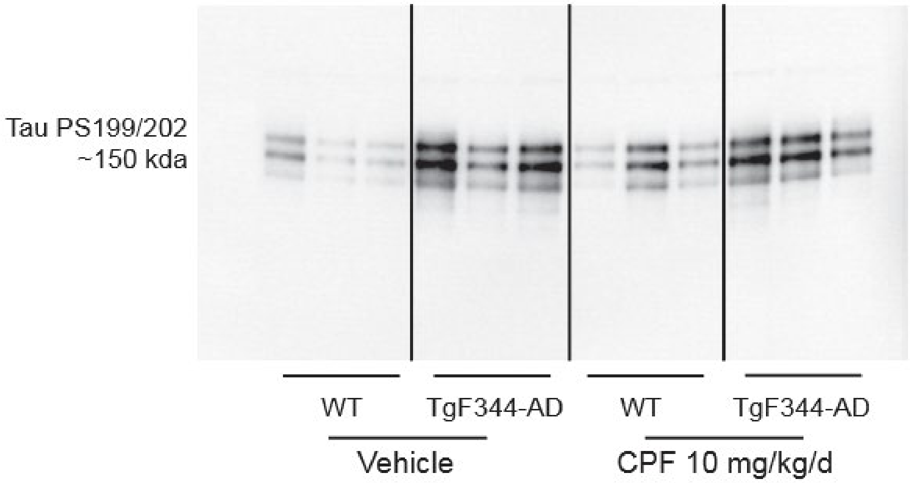

Fig. S7 Abnormally phosphorylated tau (tau$^{PS199/202}$) protein expression is elevated in TgF344-AD rats regardless of CPF-exposure. WT and TgF344-AD total tau protein is quantified using western blot analysis in rats treated with vehicle or CPF 10 mg/kg/d. Equal volumes of isolated sarkosyl soluble tau fractions were loaded and the quantification is represented in Figure 5F.

Supplementary Fig 8.

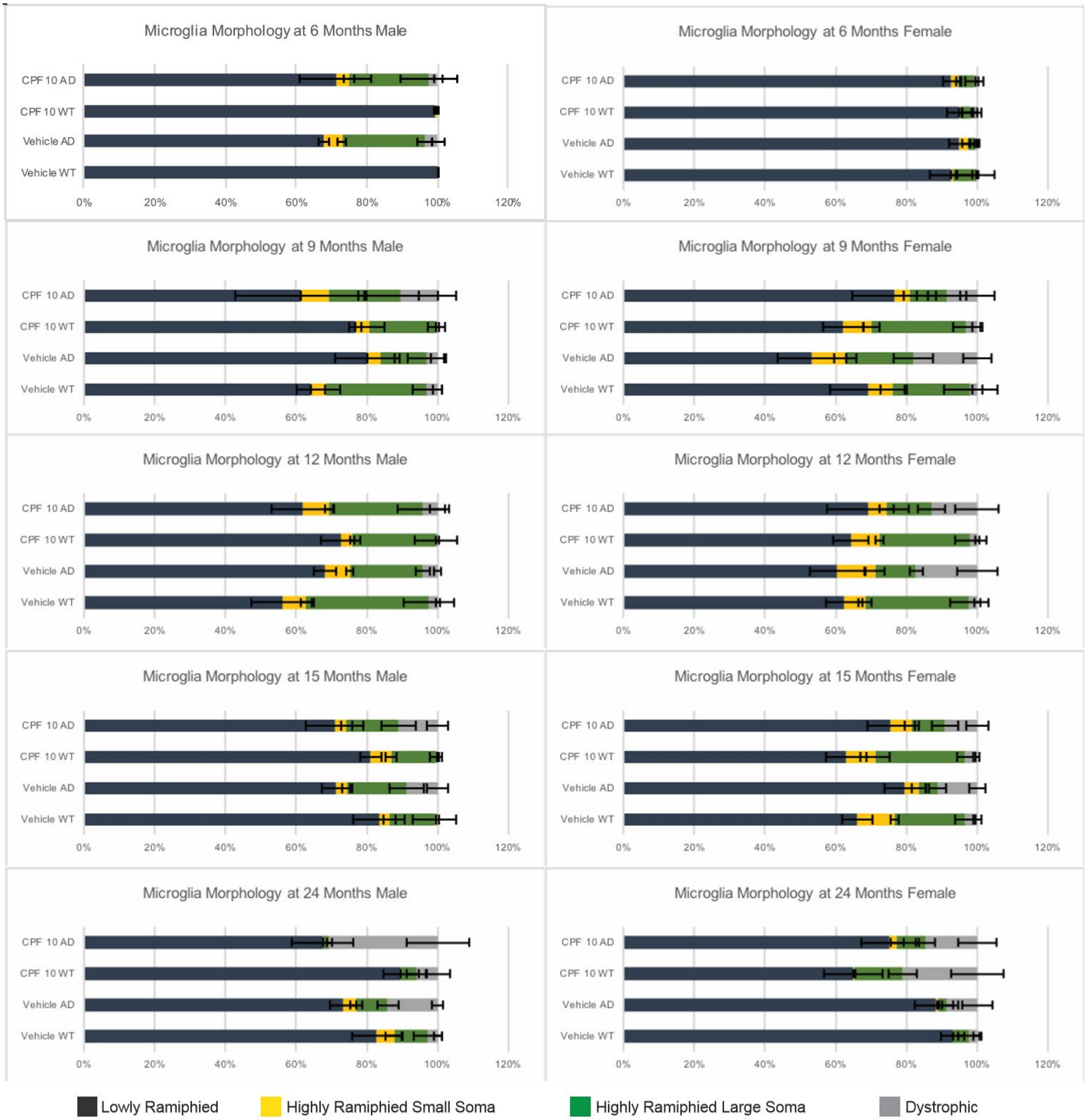

Fig. S8 CPF exposure does not significantly impact microglia morphology in the hippocampus longitudinally. Microglia cell morphology changes longitudinally, but is unaffected by CPF exposure. Male WT and TgF344-AD microglia populations are distinct at 6 months of age, but become more similar with age. Male and female microglia morphologies are also distinct at 6 months of age, but become more similar with age.

Supplemental Table 1. Serum analytes indicative of a peripheral immune response are not significantly affected by CPF exposure, transgene, or age.

	Analyte	Range (pg/mL)	Number of Samples within Detection Range (out of a total of 4)							
			Male				Female			
			Vehicle		CPF 10 mg/kg/d		Vehicle		CPF 10 mg/kg/d	
			WT	TgF344-AD	WT	TgF344-AD	WT	TgF344-AD	WT	TgF344-AD
6 months	G-CSF	2.95 - 12100	1	0	1	0	0	0	0	1
9 months			0	0	0	0	0	0	1	0
12 months			1	1	0	0	0	0	1	2
15 months			0	0	1	0	0	1	1	0
24 months			2	1	0	1	0	0	0	1
6 months	GM-CSF	4.86 - 19900	0	0	0	0	0	0	0	0
9 months			0	0	0	0	0	0	0	0
12 months			0	0	0	0	0	0	0	0
15 months			0	0	0	0	0	0	0	0
24 months			0	0	0	0	0	0	0	0
6 months	INF-γ	4.35 - 17800	0	0	0	0	0	0	0	0
9 months			0	0	0	0	0	0	0	0
12 months			0	0	0	0	0	1	0	0
15 months			0	0	1	0	1	0	1	0
24 months			3	3	2	3	0	0	0	2
6 months	IL-1α	12.55 - 51400	0	0	0	0	0	0	0	0
9 months			0	0	0	0	0	0	0	0
12 months			0	0	0	0	0	0	0	0
15 months			0	0	0	0	0	0	0	0
24 months			0	0	0	0	0	0	0	0
6 months	IL-1β	10.42 - 42700	0	0	0	0	0	1	0	0
9 months			0	0	0	0	0	0	0	0
12 months			0	0	0	0	1	0	2	0
15 months			0	0	0	0	0	0	1	0
24 months			1	1	0	1	0	1	1	2
6 months	IL-10	3.2 - 55700	0	0	0	0	0	0	0	0
9 months			0	0	0	0	0	0	0	0
12 months			0	0	0	0	0	0	0	0
15 months			0	0	0	0	0	0	0	0
24 months			1	0	1	0	0	0	0	0
6 months	IL-12p70	4.93 - 20200	0	0	0	0	0	0	0	0
9 months			0	0	0	0	0	0	1	0
12 months			0	0	0	0	0	0	0	0
15 months			0	0	0	0	0	0	0	0
24 months			1	0	0	0	0	0	0	0
6 months	IL-13	3.17 - 13000	0	0	0	0	0	0	0	0
9 months			0	0	0	0	0	0	0	0
12 months			0	0	0	0	0	0	0	0
15 months			0	0	0	0	0	0	0	0
24 months			0	0	0	1	0	0	0	1
6 months	IL-17A	2.61 - 10700	0	0	0	0	0	0	0	0
9 months			0	0	0	0	0	0	0	0
12 months			0	0	0	0	0	0	0	0
15 months			0	0	0	0	1	0	0	0
24 months			1	0	0	1	0	0	0	2
6 months	IL-2	2.1 - 8600	0	0	0	0	0	0	0	0
9 months			0	0	0	0	0	0	0	0
12 months			0	0	1	0	0	0	0	0
15 months			0	0	0	0	0	0	1	0
24 months			1	0	0	1	1	1	1	0
6 months	IL-4	0.85 - 3500	0	0	0	0	0	0	1	2
9 months			0	0	0	0	0	0	0	0
12 months			0	1	0	0	0	0	0	0
15 months			0	0	0	0	0	0	0	0
24 months			2	0	2	1	0	0	0	0

6 months	IL-5	1.65 - 6750	0	0	0	0	0	0	0	0
9 months			0	0	0	0	0	0	0	0
12 months			0	1	0	1	0	0	0	0
15 months			0	0	0	0	0	0	0	0
24 months			3	1	1	1	1	1	1	0
6 months	IL-6	1.94 - 7950	0	0	0	0	0	0	0	0
9 months			0	0	0	0	0	0	0	0
12 months			0	0	0	0	0	0	0	0
15 months			0	0	0	0	0	0	0	0
24 months			1	0	0	0	0	0	0	0
6 months	TNF-α	3.08 - 12600	0	0	0	0	0	0	0	0
9 months			0	0	0	0	0	0	0	0
12 months			0	0	0	0	0	0	0	0
15 months			0	0	0	0	0	0	0	0
24 months			0	0	0	1	0	0	0	1
